# Serum cholesterol and the risk of developing hormonally driven cancers: A narrative review

**DOI:** 10.1002/cam4.5463

**Published:** 2022-11-29

**Authors:** Dana J. Murdock, Robert J. Sanchez, Kusha A. Mohammadi, Sergio Fazio, Gregory P. Geba

**Affiliations:** ^1^ Regeneron Pharmaceuticals, Inc. Tarrytown New York USA

**Keywords:** cancer, cholesterol, incident cancer, reproductive cancer, serum lipids

## Abstract

Although cholesterol has been hypothesized to promote cancer development through several potential pathways, its role in the risk of developing hormonally driven cancer is controversial. This literature review summarizes evidence from the highest quality studies to examine the consistency and strength of the relationship between serum cholesterol parameters and incidence of hormonally driven cancer. Articles were identified using EMBASE. Longitudinal observational studies published between January 2000 and December 2020 were considered for inclusion. The endpoint of interest was incident prostate, ovary, breast, endometrium, and uterine cancers. In total, 2732 reports were identified and screened; 41 studies were included in the review. No associations were found for ovarian cancer. Most endometrial cancer studies were null. The majority (76.9%) of studies reported no association between cholesterol and prostate cancer. Data on breast cancer were conflicting, associations limited, and effect sizes modest. Our results do not provide evidence for a clear association between cholesterol and different types of incident, hormonally driven reproductive cancers. Future studies should investigate the impact of lipid‐lowering therapy.

## Graphical abstract

A literature review summarizing evidence from studies examining the consistency and strength of the relationship between serum cholesterol parameters and incidence of hormonally driven cancer was conducted. The endpoint of interest was incident prostate, ovary, breast, endometrium, and uterine cancers. In total, 2372 reports were identified and screened; 41 studies were included in the review. Overall, our results do not provide evidence for a clear association between cholesterol and different types of incident, hormonally driven cancers.

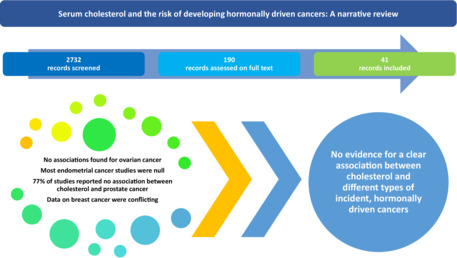

## 1 BACKGROUND

Cancer, a principal cause of mortality worldwide, was attributed to nearly 10 million deaths in 2020 alone and is the second most common cause of death in the USA after heart disease.^1^, ^2^ Cholesterol has been hypothesized to promote the development of cancer through several potential pathways. Cholesterol is a precursor molecule for corticosteroids and sex hormones, which have been shown to initiate or promote colon, prostate, and breast cancers.^3^, ^4^, ^5^ Therefore, the availability of cholesterol may influence the development and progression of hormone‐dependent tumors.^6^, ^7^, ^8^, ^9^, ^10^ Cholesterol is also required for membrane synthesis^11^ and thus it may be a limiting factor in the prolific cell division in cancer. Changes in lipid metabolism are considered a key factor in tumorigenesis,^12^ with abnormal lipid profiles being a common finding in patients with cancer. Neoplastic tissues affect blood cholesterol levels through several mechanisms, including via the elevated expression of low‐density lipoprotein receptor, the upregulation of cholesterol synthesis, and in some instances by triggering lipoprotein accumulation and cholesterol deposition.^13^ Cancer cells tend to have high cholesterol content,^14^, ^15^ with the accumulation of cholesterol identified in several different human cancers, such as breast, prostate, colon, and others.^16^, ^17^, ^18^, ^19^ Furthermore, cholesterol metabolites, such as 27‐hydroxycholesterol, have the capacity to signal through the estrogen receptor (ER) or liver X receptor, impacting breast cancer pathophysiology.^20^, ^21^ Lastly, in mouse models, the inhibition of proprotein convertase subtilisin/kexin type 9 (PCSK9), a key protein in the regulation of cholesterol metabolism, may augment immune checkpoint therapy via mechanisms involving the promotion of intratumoral T‐cell infiltration, suggesting additional potential roles for cholesterol in the tumor microenvironment which may modify (constrain) cancer growth.^22^

The role of cholesterol in the risk of developing hormonally driven cancers is debated; epidemiological and Mendelian randomization studies have shown conflicting evidence regarding the association between cholesterol and hormonally driven cancers; some support a positive association,^23^, ^24^, ^25^, ^26^, ^27^ others an inverse association or no association.^28^, ^29^, ^30^, ^31^ Reverse causation may contribute to these conflicting findings, as cancer cells are known to decrease serum cholesterol levels through several mechanisms. In addition, a long lag‐time between exposure and outcome, protopathic bias (inclusion of cancers that may be pre‐existing prior to exposure), inadequate sample sizes, the use of cross‐sectional study designs, and the inability to fully account for confounding factors may all contribute to the inconclusive evidence available to date.

The objective of this work was to summarize the available literature assessing the link between cholesterol and hormonally driven cancers. Further studies are needed to assess the role of cholesterol in the development of other cancer types.

## 2 METHODS

We conducted a literature review to find studies that assessed the association between serum cholesterol levels and the development of hormonally driven reproductive cancers in humans.

### 2.1 Eligibility criteria

Studies reporting on adults (≥18 years of age) in the general population who were at risk of developing hormonally driven cancers (and without the cancer of interest at baseline) were included. Studies enrolling both those <18 years old and adults (≥18 years old) were included if >80% of the population were adults, or if separate data were presented for adults. Studies enrolling patients who already had cancer at the start of the study were excluded. The exposure of interest was high serum cholesterol levels versus low serum cholesterol levels. High versus low cholesterol could be assessed incrementally (tertiles and quartiles) or using a single cutoff point. Cholesterol measurements of interest included total cholesterol, high‐density lipoprotein cholesterol (HDL‐C), and low‐density lipoprotein cholesterol (LDL‐C). Outcomes of interest were the incidences of breast, endometrial, or ovarian cancer (for women) and the incidence of prostate cancer (for men). Studies reporting on the risk of testicular cancer in men or cervical cancer in women were excluded, since hormone levels have not been previously associated with the incidence of these cancers. Observational studies designed to assess the temporal association between cholesterol and cancer development were considered, including cohort, case‐cohort, nested case–control, and Mendelian randomization studies. Both prospective and retrospective study designs were considered. Published systematic literature reviews and/or meta‐analyses of studies with relevant designs, as noted above, were also included. The following study designs were excluded: case control studies, case series, and case reports. Interventional studies (i.e., trials) were excluded, due to additional effects that may be independent from lipid lowering. All articles and abstracts reviewed were required to be in English. Non‐English papers (even if the abstract was in English) were excluded.

### 2.2 Literature databases and search strategy

We searched the EMBASE electronic bibliographic database from January 1, 2000, through to December 14, 2020. In addition, we searched congress abstracts listed in EMBASE over a 2‐year period (January 1, 2019, through to December 14, 2020). A full‐text screen was performed on the references identified from the title and abstract screening. Systematic reviews and meta‐analyses were identified during the first pass, and manual bibliographic searching was conducted to identify any additional relevant studies. The search criteria are provided in Table S1. In brief, articles relating to cholesterol, HDL‐C, or LDL‐C and prostate, ovary, breast, endometrium, or uterus with cancer, carcinoma, tumor, malignancy, or neoplasm terms were identified.

### 2.3 Screening and abstraction

The identified reports, based on the above eligibility criteria, were screened in a two‐step process: (1) title/abstract screening and (2) full‐text screening. Two researchers performed the screening process, with each record screened once by one of the two reviewers. After removing duplicates, citations were screened for inclusion using Microsoft Excel workbooks (Microsoft Corporation, Redmond, WA). Reasons provided for inclusion/exclusion of studies were documented during both stages of screening. Citations were excluded for the following reasons: non‐English, non‐human study, incorrect patient population, no comparison of cholesterol groups, outcome of interest not reported, incorrect study design, and other. The results of the full‐text screen and abstraction were assessed by a second independent reviewer for inclusion in the review. All relevant information was extracted and summarized manually; a final quality‐control step was completed to ensure the accuracy of all extracted information summarized in the review. Relevant statistical measures abstracted included relative risk (RR), odds ratio (OR), hazard ratio (HR), or incident rates, as well as the corresponding 95% confidence intervals (CI) and *p* values.

## 3 RESULTS

### 3.1 Overview of records identified by the literature review

A flow diagram of the screening results is shown in Figure S1. In total, 2732 records were identified and screened, of which 41 references passed both screening steps and were included in the review. An overview of the 41 studies is presented in Table S2 (longitudinal studies) and Table S3 (Mendelian randomization). A summary of the number and directionality of studies by cancer type is presented in Table 1.

**TABLE 1:** Summary of findings across studies by cancer type

	Number of studies	Total cholesterol	LDL‐C	HDL‐C
Prostate cancer				
Longitudinal observational studies	17	↑ ↑ ↓ Ꝋ Ꝋ Ꝋ Ꝋ Ꝋ Ꝋ Ꝋ Ꝋ Ꝋ Ꝋ	Ꝋ Ꝋ Ꝋ	↓ Ꝋ Ꝋ Ꝋ Ꝋ Ꝋ Ꝋ
Mendelian randomization studies	3	na	Ꝋ Ꝋ Ꝋ	Ꝋ Ꝋ Ꝋ
Breast cancer				
Longitudinal observational studies	17	↑ ↑ ↓ ↓ ↓ Ꝋ Ꝋ Ꝋ Ꝋ Ꝋ Ꝋ Ꝋ	Ꝋ Ꝋ Ꝋ Ꝋ Ꝋ	↓ ↓ ↓ ↓ ↓ ↓^a^ Ꝋ Ꝋ Ꝋ Ꝋ Ꝋ Ꝋ
Mendelian randomization studies	4	Ꝋ	↑↑ Ꝋ Ꝋ	↑↑ Ꝋ Ꝋ
Endometrial cancer				
Longitudinal observational studies	5	Ꝋ Ꝋ Ꝋ Ꝋ Ꝋ^b^	Ꝋ Ꝋ Ꝋ Ꝋ	Ꝋ Ꝋ Ꝋ Ꝋ
Mendelian randomization studies	1	na	↓	Ꝋ
Ovarian cancer				
Longitudinal observational studies	2	Ꝋ Ꝋ	Ꝋ Ꝋ	Ꝋ Ꝋ
Mendelian randomization studies	0	na	na	na

Abbreviations: ↑, Higher levels of lipids were associated with an increased risk of disease; ↓, Higher levels of lipids were associated with a lower risk of disease; Ꝋ, No association between lipid and risk of disease; HDL‐C, high‐density lipoprotein cholesterol; LDL‐C, low‐density lipoprotein cholesterol; na, No studies were identified.

^a^ Significant finding among premenopausal women only.^63^

^b^ An association between total cholesterol and risk of endometrial cancer risk was observed when analyzed by quartiles of total cholesterol, but not when using medical cutoff points for total cholesterol.^74^

### 3.2 Prostate cancer findings across longitudinal studies

In total, 17 longitudinal studies reporting on the incidence of prostate cancer met the inclusion criteria, including cohorts from Korea, Scandinavia, Western Europe, the USA, and the UK (Table S2). A summary of the outcomes reported is presented in Table 2. The association between total serum cholesterol levels and prostate cancer was reported in 13 studies; the majority (10/13; 76.9%) reported no association between total cholesterol levels (assessed by quartiles, quintiles, and clinically relevant cutoff points) and risk of incident prostate cancer.^24^, ^32^, ^33^, ^34^, ^35^, ^36^, ^37^, ^38^, ^39^, ^40^ In contrast, two studies (2/13; 15.4%) reported a positive association between total serum cholesterol and the incidence of prostate cancer,^41^, ^42^ and a single study reported an inverse association between total serum cholesterol and the incidence of prostate cancer.^43^ The largest prospective cohort study (756,604 adults; 2490 cases of incident prostate cancer) showed that higher versus lower total cholesterol levels (200–239 mg/dl and ≥240 mg/dl vs. <160 mg/dl [reference group]) were positively associated with prostate cancer (HR [95% CI]: 1.14 [1.01–1.29] and 1.24 [1.07–1.44]), respectively.^41^ The association remained after excluding the first 5 years of follow‐up. A prospective, population‐based study among Dutch men who had never used lipid‐lowering therapies (*n* = 2118; 43 cases) reported a positive association between higher total cholesterol and an increased risk of prostate cancer (per mmol/L increase, HR [95% CI]: 1.39 [1.03–1.88]).^42^ Although the prospective cohort study of 12,926 men (650 cases) who were enrolled in the Midspan studies found no association between high cholesterol and overall risk of prostate cancer (assessed by quintiles), an association between high cholesterol and risk of developing high‐grade prostate cancer (Gleason score ≥8) was observed (Q4 vs. Q1 [6.1–6.69 vs. < 5.05 mmol/L], HR [95% CI]: 2.28 [1.27–4.10]).

**TABLE 2:** Summary of outcomes reported for prostate cancer

Reference	Longitudinal Studies						
	Study design	Location	Cohort size	Age^a^	Number of cases	Exposure	Outcome
Grundmark et al. (2010)^45^	Prospective, longitudinal study	Uppsala, Sweden	2322	50 years	237	HDL‐C < 1.03 mmol/L High triglycerides, ≥1.7 mmol/L	Low HDL‐C and high triglycerides were not associated with prostate cancer Low HDL‐C: RR (95% CI): 0.95 (0.65–1.4) High triglycerides: RR (95% CI): 1.15 (0.89–1.48)
Häggström et al. (2012)^39^	Prospective, cohort study	Norway, Sweden, Austria	289,866	Mean (SD): 44 (11) years	6673	Cholesterol, quintiles, mmol/L, mean (SD): Q1: 4.28 (0.49) Q2: 5.12 (0.28) Q3: 5.68 (0.28) Q4: 6.29 (0.31) Q5: 7.43 (0.82)	No association between high cholesterol and risk of prostate cancer Cox regression models adjusted for smoking status, BMI, subcohort, birth cohort, and age at measurement Q5 versus Q1: RR (95% CI): 0.96 (0.85–1.10); *p*_trend_ 0.711
						Cholesterol, divided into quintiles and transformed to a standardized score (z score)	Model 1 (adjusted for smoking status, BMI, birth cohort, and age at measurement): RR (95% CI): 1.00 (0.96–1.04) Model 2 (adjusted for smoking status, birth cohort, age at measurement, and for z scores of all separate metabolic exposures [BMI, blood pressure, glucose, and triglycerides]): RR (95% CI): 1.02 (0.98–1.07) Subgroup analysis according to calendar period: Follow‐up until December 31, 1996: RR (95% CI): 1.01 (0.92–1.10) Follow‐up from January 1, 1997: RR (95% CI): 1.00 (0.96–1.05)
Håheim et al. (2006)^40^	Prospective, cohort study	Oslo, Norway	15,933	40–49 years	507	Total serum cholesterol, increasing Total cholesterol at screening, mmol/L, mean (SD): Cases: 6.38 (1.09) Controls: 6.38 (1.17)	No association found between increasing cholesterol and risk of prostate cancer Age‐adjusted univariate analysis: RR per 1 mmol/L increase (95% CI): 1.02 (0.94–1.10)
Heir et al. (2016)^43^	Prospective, cohort study	Oslo, Norway	1997	40–59 years	213	Total cholesterol, quartiles, mmol/L: Q1: 2.7–5.8 Q2: 5.9–6.6 Q3: 6.7–7.4 Q4: 7.5–15.4	Low cholesterol was associated with increased risk of prostate cancer Analyzed as a competing risk model Full‐time follow‐up period, univariate analysis, SHR (95% CI): Q1: 2.15 (1.44–3.22); Q2: 1.36 (0.88–2.09); Q3: 1.42 (0.92–2.21); Q4: 1 (reference); *p*_trend_ < 0.001 Full‐time follow‐up period, multivariate analysis (adjusted for age, smoking, physical fitness, BMI, and systolic blood pressure), SHR (95% CI): Q1: 2.00 (1.32–3.03); Q2: 1.29 (0.83–2.00); Q3: 1.36 (0.87–2.12); Q4: 1 (reference); *p*_trend_ < 0.001 Restricted to >20 years follow‐up, multivariate analysis (adjusted for age, smoking, physical fitness, BMI, and systolic blood pressure), SHR (95% CI): Q1: 2.00 (1.20–3.34); Q2: 1.46 (0.86–2.48); Q3: 1.55 (0.90–2.66); Q4: 1 (reference); *p*_trend_ 0.01
His et al. (2014)^33^	Prospective study of RCT cohort	France	7557	Mean (SD): 48.8 (6.3) years (non‐cases) 54.7 (4.7) years (cases)	128	Total cholesterol, HDL‐C, and LDL‐C	Total cholesterol, LDL‐C, or HDL‐C were not associated with prostate cancer risk Model 1 was adjusted for: age; intervention group; number of dietary records; height; BMI; physical activity; smoking status; educational level; alcohol intake; energy intake; history of cancer (first‐degree relatives); fasting glycemia, triglycerides; family history of prostate cancer; and baseline PSA levels Model 2 was adjusted for the same covariates as Model 1 AND use of medical treatment (blood glucose‐lowering drugs, lipid‐lowering therapies, triglyceride‐lowering drugs, and antihypertensive drugs)
						Total cholesterol, mmol/L; cutoffs for quartiles were 5.53, 6.13, and 6.80	Model 1, quartiles, HR (95% CI): Q4 versus Q1: 0.99 (0.56–1.73); *p*_trend_ 0.9 Model 1, continuous per one‐unit increment, HR (95% CI): 1.00 (0.83–1.22); *p*_trend_ 0.97 Model 2, quartiles, HR (95% CI): Q4 versus Q1: 0.99 (0.57–1.73); *p*_trend_ 0.9 Model 2, continuous per one‐unit increment, HR (95% CI): 1.01 (0.83–1.22); *p*_trend_ 0.96
						LDL‐C, mmol/L; cutoffs for quartiles were 3.39, 3.86, and 4.35	Model 1, quartiles, HR (95% CI): Q4 versus Q1: 1.40 (0.80–2.44); *p*_trend_ 0.5 Model 1, continuous per one‐unit increment, HR (95% CI): 1.07 (0.84–1.37); *p*_trend_ 0.6 Model 2, quartiles, HR (95% CI): Q4 versus Q1: 1.40 (0.80–2.44); *p*_trend_ 0.5 Model 2, continuous per one‐unit increment, HR (95% CI): 1.07 (0.84–1.37); *p*_trend_ 0.6
						HDL‐C, mmol/L; cutoffs for quartiles were 1.51, 1.71, and 1.91	Model 1, quartiles, HR (95% CI): Q4 versus Q1: 0.70 (0.39–1.25); *p*_trend_ 0.1 Model 1, continuous per one‐unit increment, HR (95% CI): 0.68 (0.36–1.26); *p*_trend_ 0.2 Model 2, quartiles, HR (95% CI): Q4 versus Q1: 0.70 (0.39–1.25); *p*_trend_ 0.1 Model 2, continuous per one‐unit increment, HR (95% CI): 0.68 (0.36–1.27); *p*_trend_ 0.2
Kitahara et al. (2011)^41^	Prospective, cohort study	Korea	756,604	44.9 years	2490	Total cholesterol, mg/dl, cutoff points: <160 (low) 160–179 180–199 200–239 ≥240 (high)	High versus low cholesterol was positively associated with risk of prostate cancer in men. ≥240 mg/dl versus <160 mg/dl (model adjusted for smoking status, alcohol status, BMI, fasting serum glucose, hypertension, and physical activity): HR (95% CI): 1.24 (1.07–1.44)
Kok et al. (2011)^42^	Prospective, population‐based study	The Netherlands	2118	Median (IQR): 62.0 (47.3–73.1) years	43	Total cholesterol, HDL‐C, and LDL‐C, per mmol/L increase	Total cholesterol and LDL were associated with an increased risk of total prostate cancer
						Total cholesterol, per mmol/L increase	Model adjusted for age, BMI, and history of diabetes mellitus (self‐reported): HR (95% CI): 1.39 (1.03–1.88)
						HDL‐C, per mmol/L increase	Model adjusted for age, BMI, and history of diabetes mellitus (self‐reported): HR (95% CI): 2.16 (0.92–5.05)
						LDL‐C, per mmol/L increase	Model adjusted for age, BMI, and history of diabetes mellitus (self‐reported): HR (95% CI): 1.42 (1.00–2.02)
Martin et al. (2009)^34^	Prospective, cohort study	Norway	29,364	Mean (SD): 48.0 (16.4) years (without MetS) 53.4 (16.3) years (with MetS)	687	Total cholesterol, HDL‐C	No association between MetS or its components and prostate cancer risk Model adjusted for age, height, smoking status, marital status, education, physical activity, and IPPS
						Total cholesterol, mmol/L, quartiles: Q1: <5.0 Q2: 5.1–5.8 Q3: 5.9–6.6 Q4: ≥6.7 Total cholesterol, mmol/L, per SD (1.2 mmol/L)	By quartiles, Q4 versus Q1: HR (95% CI): 1.10 (0.87–1.40); *p* = 0.39 Per SD (1.2 mmol/L): HR (95% CI): 1.05 (0.97–1.14); *p* = 0.24
						HDL‐C, mmol/L, quartiles: Q1: <1.1 Q2: 1.1–1.2 Q3: 1.3–1.4 Q4: ≥1.5 HDL‐C, mmol/L, per SD (0.3 mmol/L)	By quartiles, Q4 versus Q1: HR (95% CI): 0.93 (0.76–1.14); *p* = 0.66 Per SD (0.3 mmol/L): HR (95% CI): 0.98 (0.91–1.05); *p* 0.56
Mondul et al. (2010)^35^	Prospective study	Washington County, MD	6816	Mean: 53.9–54.8 years	438	Total cholesterol, mg/dl, clinical cutoff points: Desirable: <200 Borderline: 200–239 High: ≥240	Cholesterol was not associated with total prostate cancer risk. Results suggest cholesterol influences risk of high‐grade prostate cancer Model adjusted for: age; race; education level; smoking status; BMI; intake of meat, dairy products, and tomato products; alcohol intake; family history of prostate cancer; history of PSA screening; and use of diabetes medications Total cholesterol cutoff points, HR (95% CI): <200 mg/dl: 1.06 (0.81–1.39) 200–239 mg/dl: 0.93 (0.70–1.22) ≥240 mg/dl: 1.0 (reference); *p*_trend_ 0.47
Monroy‐Iglesias et al. (2021)^46^	Prospective cohort study	UK	220,622	Mean: 56 years	5409	Low HDL‐C, ≤ 1.0 mmol/L	MetS had no association with prostate cancer risk. HDL as an individual component to MetS did not show an association with prostate cancer risk Model 1 (adjusted for history of father with prostate cancer; socioeconomic status, and history of PSA testing), HDL ≤1.0 mmol/L: HR (95% CI): 0.92 (0.93–0.99) Model 2 (adjusted for history of father with prostate cancer; socioeconomic status, history of PSA testing, ethnicity, history of other cancer, metformin use, statin use, BMI, physical activity, and smoking status), HDL ≤1.0 mmol/L: HR (95% CI): 0.99 (0.91–1.06)
Pedersen et al. (2020)^47^	Prospective cohort study	Copenhagen, Denmark	Copenhagen General Population Study (*n* = 47,283)	≥20 years	1607	HDL‐C, mg/dl, cutoff points: <39 39–58 58–77 ≥77 HDL‐C, per SD decrease (19 mg/dl)	Low HDL‐C was not associated with prostate cancer risk Multivariate analysis (adjusted for age, BMI, smoking status, cumulative tobacco consumption, alcohol intake, physical activity, education, income, plasma triglycerides, lipid‐lowering therapy, C‐reactive protein, and baseline chronic disease), HR (95% CI): <39 mg/dl: 1.00 (0.73–1.35) 39–58 mg/dl: 1.04 (0.82–1.31) 58–77 mg/dl: 1.11 (0.88–1.39) ≥77 mg/dl: 1 (reference); *p*_trend_ 0.86 Multivariate analysis (adjusted for age, BMI, smoking status, cumulative tobacco consumption, alcohol intake, physical activity, education, income, plasma triglycerides, lipid‐lowering therapy, C‐reactive protein, and baseline chronic disease), HR (95% CI): per 19 mg/dl (1 SD) decrease: 1.02 (0.93–1.11); *p* = 0.74
Platz et al. (2008)^36^	Prospective, nested case–control study	USA	18,018	40–75 years	Cases: 698 Controls: 698	Cholesterol, mg/dl, Q1 versus above; cutoff points used for each analytical batch (year): 1996: 192.8 mg/dl 1998: 185.7 mg/dl 2000: 140.1 mg/dl Cholesterol, mg/dl, quartiles	Low cholesterol (< or ≥25th percentile) was not associated with prostate cancer risk Logistic regression model excluding men with a history of cholesterol‐lowering therapy use, adjusted for: age; PSA test before blood draw; family history of prostate cancer; height; vigorous physical activity; diabetes mellitus; vasectomy; cigarette smoking in the past 10 years; intake of energy, red meat, fish, and alcohol; and intake of energy‐adjusted lycopene, calcium, fructose, α‐linolenic acid; and supplemental vitamin E and selenium Cholesterol, Q1 versus above (cutoff points differed by analytical batch), OR (95% CI): 0.93 (0.72–1.21) Cholesterol, by quartiles, OR (95% CI): Q1: 1.00 (reference) Q2: 1.02 (0.74–1.41) Q3: 1.15 (0.84–1.59) Q4: 1.05 (0.76–1.45); *p*_trend_ 0.60
Platz et al. (2009)^37^	Prospective cohort study	USA	5586	≥55 years	1251	Cholesterol, mg/dl, quintiles: Q1: <182 Q2: 182–200 Q3: 201–218 Q4: 219–240 Q5: ≥241 Cholesterol, mg/dl, low (normal: <200 mg/dl) versus high (borderline and elevated cholesterol: ≥200 mg/dl)	No association between cholesterol and overall prostate cancer risk Age‐adjusted analysis by quintiles, OR (95% CI): Q1: 0.93 (0.76–1.13) Q2: 0.89 (0.73–1.09) Q3: 0.90 (0.74–1.09) Q4: 1.02 (0.84–1.24) Q5: 1.00 (reference); *p*_trend_ 0.37 Multivariate analysis by quintiles (adjusted for age, race, family history of prostate cancer, BMI, diabetes, regular aspirin use, and history of heart attack), OR (95% CI): Q1: 0.94 (0.77–1.15) Q2: 0.90 (0.74–1.10) Q3: 0.90 (0.74–1.09) Q4: 1.03 (0.85–1.25) Q5: 1.00 (reference); *p*_trend_ 0.43 Age‐adjusted analysis for <200 versus ≥200 mg/dl, OR (95% CI): 0.96 (0.85–1.10) Multivariate analysis for <200 versus ≥200 mg/dl (adjusted for age, race, family history of prostate cancer, BMI, diabetes, regular aspirin use, and history of heart attack), OR (95% CI): 0.97 (0.85–1.11)
Shafique et al. (2012)^24^	Prospective cohort study	Scotland, UK	12,926	Median (range): 51 (21–75) years	650	Cholesterol, mmol/L, quintiles: Q1: <5.05 Q2: 5.06–5.57 Q3: 5.58–6.09 Q4: 6.1–<6.69 Q5: ≥6.7	High cholesterol is positively associated with a greater risk of high‐grade prostate cancer but not the overall risk of prostate cancer Unadjusted analysis for overall prostate cancer risk, HR (95% CI): Q1: 1 (reference) Q2: 0.87 (0.60–1.26) Q3: 1.03 (0.72–1.46) Q4: 1.36 (0.97–1.92) Q5: 1.09 (0.76–1.57) Multivariate analysis for overall prostate cancer risk, (adjusted for age, smoking status, BMI, and socioeconomic status), HR (95% CI): Q1: 1 (reference) Q2: 0.89 (0.70–1.14) Q3: 0.95 (0.75–1.21) Q4: 1.01 (0.79–1.29) Q5: 0.95 (0.74–1.22)
Strohmaier et al. (2013)^32^	Prospective cohort study	Norway, Austria, Sweden	289,273	Mean: 40.3–47.5 years	6884	Cholesterol, by quintiles Cholesterol, per 1 mmol/L increment	Total serum cholesterol was not associated with prostate cancer risk Models used age as the timescale, stratified by cohort, fasting status, and birth year categories, adjusted for baseline age, BMI, and smoking status Quintiles, HR (95% CI): Q1: 1 (reference) Q2: 1.00 (0.88–1.14) Q3: 1.05 (0.93–1.20) Q4: 1.01 (0.89–1.14) Q5: 0.99 (0.88–1.13); *p*_trend_ 0.86 Per 1 mmol/L increment, HR (95% CI): 0.99 (0.96–1.03)
Van Hemelrijck et al. (2011)^38^	Prospective cohort study	Sweden	200,660	45–75 years	5112	Total cholesterol, mmol/L, quartiles: Q1: <5.1 Q2: 5.1–5.8 Q3: 5.8–6.5 Q4: >6.5	No association between hypercholesterolemia (total cholesterol ≥6.5 mmol/L) and prostate cancer risk Overall population, quartiles, HR (95% CI): Q1: 1.00 (reference) Q2: 1.08 (0.99–1.17) Q3: 1.06 (0.98–1.15) Q4: 0.99 (0.91–1.08); *p*_trend_ 0.583
Van Hemelrijck et al. (2011)^44^	Prospective cohort study	Sweden	69,735	Mean (SD): 59.66 (8.86) years (cases) 50.23 (9.98) years (non‐cases)	2008	HDL‐C and LDL‐C, by quartiles	Low HDL‐C levels are related to increased prostate cancer risk LDL‐C and non‐HDL‐C were not associated with prostate cancer risk Models stratified by age, and adjusted for glucose, triglycerides, fasting status, and socioeconomic status
						HDL‐C, mmol/L, by quartiles: Q1: <1.13 Q2: 1.13–1.39 Q3: 1.39–1.65 Q4: >1.65	Total cohort, HR (95% CI): Q1: 1.00 (reference) Q2: 0.93 (0.81–1.07) Q3: 0.88 (0.76–1.01) Q4: 0.81 (0.70–0.94); *p*_trend_ 0.004 Excluding first 3 years of follow‐up, HR (95% CI): Q1: 1.00 (reference) Q2: 0.89 (0.78–1.03) Q3: 0.84 (0.73–0.98) Q4: 0.79 (0.68–0.92); *p*_trend_ 0.003
						LDL‐C, mmol/L, by quartiles: Q1: <3.19 Q2: 3.19–3.83 Q3: 3.83–4.52 Q4: >4.52	Total cohort, HR (95% CI): Q1: 1.00 (reference) Q2: 0.96 (0.85–1.09) Q3: 1.03 (0.90–1.16) Q4: 1.02 (0.90–1.16); *p*_trend_ 0.495 Excluding first 3 years of follow‐up, HR (95% CI): Q1: 1.00 (reference) Q2: 0.97 (0.84–1.11) Q3: 1.03 (0.90–1.18) Q4: 1.05 (0.92–1.20); *p*_trend_ 0.308

Reference	Mendelian Randomization Studies		
	Country/Region	Study name/cohort	Main conclusions/outcomes
Adams et al. (2019)^50^	UK	GWAS: Kettunen et al. (2016)^78^ and PRACTICAL consortium^79^ Risk analysis: ProtecT trial (median age at baseline: 63 years) Cases: *n* = 2291 Controls: *n* = 2661	Thirty‐five circulating metabolites associated with prostate cancer presence were identified, but there was no evidence of causality for the 14 that were testable with MR Causal estimates from MR for cholesterol metabolites included: Cholesterol esters in medium LDL: OR (95% CI): 0.976 (0.927–1.028); *p* = 0.363 Free cholesterol in IDL: OR (95% CI): 1.026 (0.973–1.082); *p* = 0.339 Free cholesterol in medium HDL: OR (95% CI): 1.004 (0.896–1.125); *p* = 0.947 Total cholesterol in medium LDL: OR (95% CI): 0.979 (0.930–1.031); *p* = 0.422 Free cholesterol in large LDL: OR (95% CI): 1.000 (0.950–1.060); *p* = 0.860 Total cholesterol in small LDL: OR (95% CI): 0.967 (0.912–1.025); *p* = 0.256 Total lipids in small HDL: OR (95% CI): 1.020 (0.930–1.120); *p* = 0.690 Phospholipids in IDL: OR (95% CI): 1.016 (0.962–1.072); *p* = 0.572 Phospholipids in very small VLDL: OR (95% CI): 1.022 (0.960–1.087); *p* = 0.501 Causal estimates from MR for other related metabolites included: Lipoprotein subclass particles, concentration of small HDL: OR (95% CI): 1.017 (0.914–1.131); *p* = 0.764 Lipoprotein particle size, VLDL: OR (95% CI): 1.081 (0.996–1.173); *p* = 0.061
Bull et al. (2016)^48^	Australia, Bulgaria, Denmark, Europe, Finland, Germany, Poland, Portugal, Sweden, the USA, and the UK	GWAS: Do et al. (2013)^80^; Teslovich et al. (2010)^81^; Global Lipids Genetics Consortium^76^; Isaacs et al. (2013)^82^ Risk analysis: PRACTICAL consortium Cases: *n* = 22,249 (mean age at diagnosis: 54.0–70.0 years) Controls: *n* = 22,133	Circulating lipids (proxied by genetic risk scores) did not appear to alter prostate cancer risk Meta‐analysis OR prostate cancer per unit increase in genetic risk scores (SD trait): LDL: OR (95% CI): 1.24 (0.90–1.69); *p* = 0.18 HDL: OR (95% CI): 0.99 (0.84–1.17); *p* = 0.90
Orho‐Melander et al. (2018)^49^	Sweden	Malmö Diet and Cancer Study (*n* = 26,904; mean [SD] age at baseline: 57.8 [7.6] years) Prostate cancer cases: *n* = 1322 GWAS for lipids: Teslovich TM et al. Biological, clinical and population relevance of 95 loci for blood lipids^81^	LDL IV and HDL IV were not related to incident risk of prostate cancer in logistic regression analysis or in multivariable pleiotropy‐adjusted analysis **Prostate cancer** LDL IV, per SD increment: OR (95% CI): 0.96 (0.78–1.19) LDL IV multivariable, per SD increment: OR (95% CI): 1.04 (0.87–1.24) LDL serum, per SD increment: OR (95% CI): 1.05 (0.92–1.20) HDL IV, per SD increment: OR (95% CI): 0.93 (0.74–1.18) HDL IV multivariable, per SD increment: OR (95% CI): 0.93 (0.78–1.10) HDL serum, per SD increment: OR (95% CI): 0.89 (0.78–1.03)

Abbreviations: BMI, body mass index; CI, confidence interval; GWAS, genome‐wide association study; HDL‐C, high‐density lipoprotein cholesterol; HR, hazard ratio; IDL, intermediate‐density lipoprotein; IPPS, International Prostate Symptom Score; IQR, interquartile range; IV, instrument variable; LDL‐C, low‐density lipoprotein cholesterol; MetS, metabolic syndrome; MR, Mendelian randomization; OR, odds ratio; PRACTICAL, Prostate Cancer Association Group to Investigate Cancer Associated Alterations in the Genome; ProtecT, Prostate testing for cancer and Treatment; PSA, prostate‐specific antigen; RR, relative risk; SD, standard deviation; SHR, sub‐hazard ratio; VLDL, very‐low‐density lipoprotein.

^a^ Age at baseline or recruitment.

Only three studies assessed the association between LDL‐C and incidence of prostate cancer.^33^, ^42^, ^44^ None of these publications found an association between LDL‐C levels (assessed by quartiles and per mmol/L increase) and risk of prostate cancer. Although non‐significant, two studies reported estimates that trended toward an association between higher LDL‐C and increased risk of prostate cancer.^33^, ^44^ Interestingly, in a small study (only 40 cases), an association between higher levels of LDL‐C and aggressive prostate cancer was found (HR [95% CI]: 1.83 [95%CI: 1.15–2.90]) in men with triglyceride (TG) levels ≤4.52 mmol.^42^

Lastly, the association between low HDL‐C levels and prostate cancer was reported in seven studies^33^, ^34^, ^42^, ^44^, ^45^, ^46^, ^47^; the majority (6/7) reported no association between HDL‐C levels (assessed by quartiles, per standard deviation [SD] and per mmol/L increments, and by cutoff points) and risk of prostate cancer.^33^, ^34^, ^42^, ^45^, ^46^, ^47^ The studies were heterogenous in sample sizes (ranging from 43 to 5409 cases) and in the variables controlled for in the models. One prospective cohort study from the Apolipoprotein MOrtality RISk (AMORIS) database reported an inverse association between serum HDL‐C levels (assessed by quartiles) and increased prostate cancer risk (Q4 vs. Q1, HR [95% CI]: 0.81 [0.70–0.94]); similar results were obtained when the first 3 years of follow‐up were excluded.^44^

### 3.3 Prostate cancer findings across Mendelian randomization studies

Three Mendelian randomization studies assessing the association between circulating lipids and prostate cancer were identified (Table 2; Table S3).^48^, ^49^, ^50^ None of the identified studies found an association with genetic markers of lipid levels and prostate cancer risk. All three studies had very large sample sizes with >1300 cases, used two sample Mendelian randomization designs, and combined single nucleotide polymorphisms (SNPs) to form genetic scores as the main predictor in the model. The number of SNPs evaluated varied in each study. The numbers of SNPs included in the genetic scores for TGs, LDL‐C, and HDL‐C were 15, 11, and 36 for the Bull et al. study,^48^ 26, 32, and 41 for the Orho‐Melander et al. study,^49^ and 9, 14, and 7 for the Adams et al. study,^50^ respectively. Notably, although Orho‐Melander et al. found no significant associations with the identified SNPs in the genetic scores for HDL‐C, LDL‐C, or TGs, they found a significant increase in the odds of prostate cancer for each copy of HMGCR rs12916 allele, which mimics the effect of statins (OR: 1.09 [95% CI: 1.01–1.18] per LDL‐C‐lowering T‐allele).

### 3.4 Breast cancer findings across longitudinal studies

The literature review identified 17 records reporting on the association between serum cholesterol levels and risk of breast cancer, including cohorts from Austria, Denmark, France, Korea, Italy, Norway, Sweden, and the USA (Table S2). A summary of the outcomes reported for breast cancer is presented in Table 3. Overall, studies evaluating various serum lipids provided conflicting evidence for breast cancer. Twelve studies evaluated the effect of total serum cholesterol levels on incident breast cancer risk; of these, seven (58.3%) reported no association,^51^, ^52^, ^53^, ^54^, ^55^, ^56^, ^57^ two (16.7%) reported a positive association,^41^, ^58^ and three (25%) reported an inverse relationship^32^, ^33^, ^59^ between total cholesterol levels and risk of breast cancer. A more limited number of studies evaluated the association between LDL‐C levels and breast cancer; of the five records, none reported an association.^33^, ^51^, ^53^, ^54^, ^56^ Lastly, 12 studies reported on the association between HDL‐C levels and breast cancer; of these, six (50%) reported no association^51^, ^53^, ^54^, ^55^, ^56^, ^60^ and six (50%) an inverse association.^33^, ^47^, ^59^, ^61^, ^62^, ^63^ Of note, the inverse association observed in the Kucharska‐Newton et al. study was limited to the premenopausal cohort; no association was found in the postmenopausal cohort.^63^

**TABLE 3:** Summary of outcomes reported for breast cancer

Reference	Longitudinal studies						
	Study design	Location	Cohort size	Age^a^	Number of cases	Exposure	Main conclusions/outcomes
Agnoli et al. (2010)^61^	Prospective, nested case–control study	Italy	3966	Mean (SD): 57.3 (5.8) years (without MetS) 58.8 (5.5) years (with MetS)	163 (629 controls)	HDL‐C ≤ 55 mg/dl versus >55 mg/dl	Among individual MetS components, only low serum HDL‐C and high TGs were associated with increased risk of breast cancer in postmenopausal women Model adjusted for age, age at menarche, years from menopause, number of full‐term pregnancies, age at first birth, oral contraceptive use, hormone therapy, years of education, history of breast cancer in first degree relative, breastfeeding, smoking, and alcohol consumption HDL‐C ≤ 55 mg/dl, RR (95% CI): No: 1 (reference) Yes: 1.60 (1.10–2.33)
Chandler et al. (2016)^51^	Prospective, cohort study	USA	15,602	≥45 years	864	Total cholesterol, HDL‐C, and LDL‐C	Total cholesterol, HDL‐C, and LDL‐C were not associated with breast cancer risk Multivariate models adjusted for Women's Health Study treatment assignment (aspirin, vitamin E, or β‐carotene), age, cigarette smoking, exercise, alcohol consumption, postmenopausal status, postmenopausal hormone use, family history of cancer, aspirin use, and mammogram screening An additional model also included adjustment for BMI
						Total cholesterol, mg/dl, quartiles: Q1: 72.0–180.0 Q2: 181.0–205.0 Q3: 206.0–234.0 Q4: 235.0–474.0	Adjusted for age and treatment only, HR (95% CI): Q1: 1.00 (reference) Q2: 1.03 (0.85–1.25) Q3: 1.13 (0.94–1.37) Q4: 0.98 (0.80–1.19), *p*_trend_ 0.96 Multivariate model, HR (95% CI): Q1: 1.00 (reference) Q2: 1.04 (0.85–1.27) Q3: 1.17 (0.96–1.42) Q4: 1.02 (0.83–1.26), *p*_trend_ 0.67 Multivariate model including BMI, HR (95% CI): Q1: 1.00 (reference) Q2: 1.03 (0.85–1.26) Q3: 1.15 (0.95–1.40) Q4: 1.01 (0.82–1.24), *p*_trend_ 0.80
						Total cholesterol, per 1 SD increase (209.2 mg/dl)	Multivariable HR (95% CI): 0.96 (0.89–1.03)
						HDL‐C, mg/dl, quartiles: Q1: 17.1–41.3 Q2: 41.4–49.2 Q3: 49.3–58.9 Q4: 59.0–151.1	Adjusted for age and treatment only, HR (95% CI): Q1: 1.00 (reference) Q2: 1.07 (0.88–1.29) Q3: 1.08 (0.89–1.30) Q4: 0.98 (0.81–1.19), *p*_trend_ 0.76 Multivariate model, HR (95% CI): Q1: 1.00 (reference) Q2: 1.02 (0.84–1.24) Q3: 1.05 (0.86–1.28) Q4: 0.92 (0.75–1.13), *p*_trend_ 0.42 Multivariate model including BMI, HR (95% CI): Q1: 1.00 (reference) Q2: 1.03 (0.85–1.25) Q3: 1.08 (0.88–1.32) Q4: 0.96 (0.78–1.19), *p*_trend_ 0.72
						HDL‐C, per 1 SD increase (51.1 mg/dl)	HR (95% CI): 0.96 (0.89–1.04)
						LDL‐C, mg/dl, quartiles: Q1: 17.3–102.0 Q2: 102.1–123.3 Q3: 123.4–146.9 Q4: 147.0–335.4	Adjusted for age and treatment only, HR (95% CI): Q1: 1.00 (reference) Q2: 1.09 (0.91–1.32) Q3: 0.99 (0.82–1.21) Q4: 0.96 (0.78–1.16), *p*_trend_ 0.44 Multivariate model, HR (95% CI): Q1: 1.00 (reference) Q2: 1.10 (0.91–1.34) Q3: 1.03 (0.84–1.25) Q4: 1.01 (0.83–1.24), *p*_trend_ 0.90 Multivariate model including BMI, HR (95% CI): Q1: 1.00 (reference) Q2: 1.09 (0.90–1.33) Q3: 1.01 (0.83–1.24) Q4: 0.99 (0.81–1.22), *p*_trend_ 0.74
						LDL‐C, per 1 SD increase (126.1 mg/dl)	HR (95% CI): 0.95 (0.89–1.03)
Dibaba et al. (2018^58^	Prospective, cohort study	USA	94,555	50–71 years	5380	Cholesterol, elevated (self‐reported)	Elevated cholesterol was consistently associated with overall breast cancer risk and across BMI groups Models were adjusted for age, BMI, race, physical activity, education, smoking, region, family history of breast cancer, ovary status, current hormonal therapy use, and hysterectomy Overall cohort: HR (95% CI): 1.06 (1.01–1.12) Normal BMI (≥18.5–<25 kg/m^2^) cohort: HR (95% CI): 1.13 (1.04–1.23) Overweight/obese (≥25 kg/m^2^) cohort: HR (95% CI): 1.03 (0.96–1.11) Postmenopausal cohort: HR (95% CI): 1.07 (1.01–1.13) Premenopausal cohort: HR (95% CI): 0.88 (0.65–1.19) In postmenopausal women, elevated cholesterol remained associated with increased risk of breast cancer among women with normal BMI (HR [95% CI]: 1.14 [1.05–1.24])
Eliassen et al. (2005)^52^	Prospective, cohort study	USA	71,921	42–69 years	3177	Total cholesterol (self‐reported on questionnaire in 20–30 mg/dl [0.52–0.78 mmol/L] categories): <180 mg/dl 180–199 mg/dl 200–219 mg/dl 220–239 mg/dl ≥240 mg/dl	Serum cholesterol levels were not substantially associated with breast cancer risk Also, within the subgroup of postmenopausal women who had never used postmenopausal hormones, serum cholesterol levels were not associated with breast cancer risk when analyzed by ER/PR status Multivariate analysis adjusted for time in 2‐year intervals, age, age at menarche, parity, and age at first birth, height, BMI, first‐degree family history of breast cancer, benign breast disease, alcohol consumption, physical activity, menopausal status, age at menopause, and use of postmenopausal hormones Postmenopausal cohort, relative risk (95% CI): <180: 1.00 (reference) 180–199: 1.05 (0.92–1.20) 200–219: 1.01 (0.89–1.14) 220–239: 0.90 (0.78–1.03) ≥240: 1.04 (0.91–1.17), *p*_trend_ 0.29 Premenopausal cohort, relative risk (95% CI): <180: 1.00 (reference) 180–199: 0.73 (0.48–1.09) 200–219: 0.82 (0.54–1.24) 220–239: 0.72 (0.43–1.19) ≥240: 0.94 (0.54–1.64), *p*_trend_ 0.35
Furberg et al. (2004)^59^	Population‐based screening survey	Norway	38,823	17–54 years	708	Total cholesterol and HDL‐C	Low HDL‐C, as part of MetS, was associated with increased postmenopausal breast cancer risk
						Total cholesterol, mmol/L, quartiles: Q1: <5.24 Q4: >6.82	Age‐adjusted analysis of postmenopausal breast cancer risk, RR (95% CI): Q4 versus Q1: 0.63 (0.48–0.82), *p*_trend_ 0.005
						HDL‐C, mmol/L, quartiles: Q1: <1.20 Q2: 1.20–1.40 Q3: 1.41–1.64 Q4: >1.64	Postmenopausal cohort; adjusted for multiple covariates (age, country of residence, parity, height, BMI, total serum cholesterol, recreational activity, occupational activity, and menopausal status), RR (95% CI): Q1: 1.00 (reference) Q2: 0.77 (0.59–1.00) Q3: 0.76 (0.59–0.99) Q4: 0.73 (0.55–0.95), *p*_trend_ 0.03 Postmenopausal, compound cohort; adjusted for multiple covariates (age, country of residence, parity, height, BMI, total serum cholesterol, recreational activity, occupational activity, and menopausal status), RR (95% CI): Q1: 1.00 (reference) Q2: 0.89 (0.69–1.15) Q3: 0.82 (0.64–1.07) Q4: 0.75 (0.58–0.97), *p*_trend_ 0.02 Premenopausal cohort; adjusted for multiple covariates (age, country of residence, parity, height, BMI, total serum cholesterol, recreational activity, and occupational activity), RR (95% CI): Q1: 1.00 (reference) Q2: 1.50 (0.89–2.52) Q3: 1.32 (0.77–2.26) Q4: 1.43 (0.83–2.46), *p*_trend_ 0.33 Premenopausal, compound cohort; adjusted for multiple covariates (age, country of residence, parity, height, BMI, total serum cholesterol, recreational activity, and occupational activity), RR (95% CI): Q1: 1.00 (reference) Q2: 1.13 (0.69–1.86) Q3: 1.24 (0.77–1.99) Q4: 1.44 (0.91–2.30), *p*_trend_ 0.09
						HDL‐C, quartiles (as noted above) and by BMI (<vs ≥25 kg/m^2^)	The association between serum HDL‐C and the risk of postmenopausal breast cancer was confined to overweight and obese women BMI < 25 kg/m^2^, postmenopausal, compound cohort; adjusted for multiple covariates (age, country of residence, parity, height, total serum cholesterol, recreational activity, and occupational activity), RR (95% CI): Q1: 1.00 (reference) Q2: 1.27 (0.79–2.06) Q3: 1.39 (0.89–2.20) Q4: 1.33 (0.86–2.08), *p*_trend_ 0.27 BMI ≥25 kg/m^2^, postmenopausal, compound cohort; adjusted for multiple covariates (age, country of residence, parity, height, total serum cholesterol, recreational activity, and occupational activity), RR (95% CI): Q1: 1.00 (reference) Q2: 0.76 (0.54–1.08) Q3: 0.55 (0.37–0.81) Q4: 0.43 (0.28–0.67), *p*_trend_ <0.001, *p*_interaction_ 0.001 BMI < 25 kg/m^2^, premenopausal, compound cohort; adjusted for multiple covariates (age, country of residence, parity, height, total serum cholesterol, recreational activity, and occupational activity), RR (95% CI): Q1: 1.00 (reference) Q2: 1.40 (0.73–2.66) Q3: 1.44 (0.77–2.68) Q4: 1.65 (0.90–3.02), *p*_trend_ 0.11 BMI ≥25 kg/m^2^, premenopausal, compound cohort; adjusted for multiple covariates, RR (95% CI): Q1: 1.00 (reference) Q2: 0.82 (0.36–1.86) Q3:1.06 (0.48–2.33) Q4: 1.26 (0.57–2.80), *p*_trend_ 0.46, *p*_interaction_ 0.76
His et al. (2017)^53^	Prospective, nested case–control study	France	1626	Mean (SD): 56.74 (6.32) years (controls) 56.86 (6.36) years (cases)	583 (1043 controls)	Total cholesterol, LDL‐C, and HDL‐C	No significant association was detected between any lipid biomarkers and breast cancer risk overall Risk was not modified by menopausal status Analyses adjusted for age at menarche, number of children and age at first full‐term pregnancy, menopausal status, age at menopause and current menopause hormone therapy use at blood collection, use of progesterone alone within the last 24 hours before blood collection, family history of breast cancer in first‐degree relatives, personal history of benign breast disease, daily alcohol intake, daily glycemic load, daily lipid intake, daily energy intake without alcohol, BMI, and education level
						Total cholesterol, mmol/L, per doubling of concentration (log2 scale) Total cholesterol, mmol/L, tertiles: T1: <4.87 T2: 4.87–5.55 T3: ≥5.56	Per doubling, OR (95% CI): 0.98 (0.58–1.64) Tertiles, OR (95% CI): T1: 1 (reference) T2: 0.85 (0.65–1.13) T3: 0.98 (0.74–1.30), *p* = 0.84
						HDL‐C, mmol/L, per doubling of concentration (log2 scale) HDL‐C, mmol/L, tertiles: T1: <1.72 T2: 1.72–2.62 T3: ≥2.63	Per doubling, OR (95% CI): 0.97 (0.71–1.33) Tertiles, OR (95% CI): T1: 1 (reference) T2: 1.00 (0.73–1.36) T3: 0.75 (0.49–1.15), *p* = 0.16
						LDL‐C, mmol/L, per doubling of concentration (log2 scale) LDL‐C, mmol/L, tertiles: T1: <1.82 T2:1.82–2.81 T3: ≥2.82	Per doubling, OR (95% CI): 0.99 (0.72–1.35) Tertiles, OR (95% CI): T1: 1 (reference) T2: 0.92 (0.66–1.29) T3: 0.91 (0.60–1.38), *p* = 0.68
His et al. (2014)^33^	Prospective study of RCT cohort	France	7557	Mean (SD): 48.8 (6.3) years (non‐cases) 49.5 (6.1) years (cases)	141	Total cholesterol, HDL‐C, and LDL‐C	Total cholesterol and HDL‐C were associated with decreased breast cancer risk. Associations remained significant when excluding cases diagnosed during the first 3 years of follow‐up Model 1 was adjusted for age; intervention group; number of dietary records; height; BMI; physical activity; smoking status; educational level; alcohol intake; energy intake; family history of breast cancer; menopausal status at baseline; glycemia; and TGs Model 2 was adjusted for the same covariates as Model 1 AND use of medical treatment (blood‐glucose lowering drugs, lipid‐lowering therapies, TG‐lowering drugs, and antihypertensive drugs)
						Total cholesterol, mmol/L, cut‐offs for quartiles were 5.20, 5.82, and 6.54 Total cholesterol, mmol/L, continuous per one unit increment	Model 1, quartiles, HR (95% CI): Q1: 1 (reference) Q2: 0.83 (0.52–1.35) Q3: 0.99 (0.62–1.58) Q4: 0.65 (0.39–1.10), *p*_trend_ 0.2 Model 1, continuous per one‐unit increment, HR (95% CI): 0.83 (0.69–0.99), *p*_trend_ 0.04 Model 2, quartiles, HR (95% CI): Q1: 1 (reference) Q2: 0.84 (0.52–1.35) Q3: 0.99 (0.63–1.58) Q4: 0.65 (0.39–1.10); *p*_trend_ 0.2 Model 2, continuous per one‐unit increment, HR (95% CI): 0.83 (0.69–0.99), *p =* 0.04
						LDL‐C, mmol/L, cut‐offs for quartiles were 3.11, 3.56, and 4.11 LDL‐C, mmol/L, continuous per one unit increment	Model 1, quartiles, HR (95% CI): Q1: 1 (reference) Q2: 0.75 (0.46–1.22) Q3: 1.11 (0.70–1.74) Q4: 0.65 (0.39–1.09), *p*_trend_ 0.3 Model 1, continuous per one‐unit increment, HR (95% CI): 0.83 (0.66–1.05); *p*_trend_ 0.1 Model 2, quartiles, HR (95% CI): Q1: 1 (reference) Q2: 0.75 (0.46–1.22) Q3: 1.11 (0.71–1.74) Q4: 0.65 (0.39–1.09); *p*_trend_ 0.3 Model 2, continuous per one‐unit increment, HR (95% CI): 0.83 (0.66–1.05), *p*_trend_ 0.1
						HDL‐C, mmol/L, cut‐offs for quartiles were 1.66, 1.86, and 2.07 HDL‐C, mmol/L, continuous per one unit increment	Model 1, quartiles, HR (95% CI): Q1: 1 (reference) Q2: 1.10 (0.70–1.72) Q3: 0.81 (0.50–1.30) Q4: 0.61 (0.36–1.01), *p*_trend_ 0.03 Model 1, continuous per one‐unit increment, HR (95% CI): 0.48 (0.28–0.84), *p*_trend_ 0.01 Model 2, quartiles, HR (95% CI): Q1: 1 (reference) Q2: 1.10 (0.70–1.71) Q3: 0.81 (0.50–1.30) Q4: 0.60 (0.36–1.01), *p*_trend_ 0.02 Model 2, continuous per one‐unit increment, HR (95% CI): 0.48 (0.28–0.83), *p*_trend_ 0.009
Kabat et al. (2018)^54^	Prospective study	USA	24,208	Mean (SD): 64.1 (7.3) years (non‐cases) 64.0 (7.3) years (cases)	1009	Total cholesterol, HDL‐C, and LDL‐C	Total cholesterol and LDL‐C were not associated with breast cancer risk in postmenopausal women HDL‐C showed a borderline inverse association with breast cancer risk in postmenopausal women Model adjusted for: age; smoking status; pack‐years of smoking; alcohol intake; BMI; physical activity; age at first birth; age at menarche; age at menopause; oral contraceptive use; hormone therapy; parous/nulliparous; family history of first degree relative with breast cancer; history of breast biopsy; breastfed >6 months; education; ethnicity; and allocation to the observational study component or specific arm of clinical trial.
						Total cholesterol, mg/dl, quartiles	HR (95% CI): Q1: 1 (reference) Q2: 1.05 (0.87–1.27) Q3: 1.22 (1.01–1.46) Q4: 1.07 (0.89–1.30), *p*_trend_ 0.32
						HDL‐C, mg/dl, quartiles	HR (95% CI): Q1: 1 (reference) Q2: 0.94 (0.79–1.12) Q3: 0.81 (0.67–0.98) Q4: 0.83 (0.68–1.01), *p*_trend_ 0.03 When current users of hormone therapy were excluded, the inverse association of HDL with breast cancer was strengthened (Q4 vs. Q1, HR [95% CI] 0.75 [0.59–0.93]; *p*_trend_ 0.004)
						LDL‐C, mg/dl, quartiles	HR (95% CI): Q1: 1 (reference) Q2: 1.11 (0.92–1.34) Q3: 1.19 (0.98–1.43) Q4: 1.08 (0.89–1.31), *p*_trend_ 0.07
Kitahara et al. (2011)^41^	Prospective, cohort study	Korea	433,115	Mean: 49.3 years	3805	Total cholesterol, mg/dl, cutoff points: <160 160–179 180–199 200–239 ≥240	High versus low cholesterol was positively associated with risk of breast cancer in women Models adjusted for age, cigarette smoking, alcohol consumption, BMI, fasting serum glucose, hypertension, and physical activity HR (95% CI): <160: 1 (reference) 160–179: 1.08 (0.98–1.20) 180–199: 1.12 (1.01–1.24) 200–239: 1.11 (1.01–1.23) ≥240: 1.17 (1.03–1.33), *p*_trend_ 0.03 After excluding the first 5 years of follow‐up, the association of high (≥240 mg/dl) versus low (<160 mg/dl) cholesterol with breast cancer risk was slightly stronger: HR (95% CI) 1.21 (1.04–1.41); *p*_trend_ 0.003
Kucharska‐Newton et al. (2008)^63^	Prospective study	USA	7575	Mean (SD): 53.7 (5.7) years	359	HDL‐C, mg/dl, low versus high (<50 mg/dl versus ≥50 mg/dl) HDL‐C, mg/dl, quartiles: Q1: <45.2 Q2: 45.2–55.0 Q3: 55.0–67.4 Q4: ≥67.4	Low HDL among premenopausal women may be a marker of increased breast cancer risk Analyses adjusted for age, race, BMI, smoking, age at menarche, reproductive hormone use, and age at menopause (total cohort and postmenopausal women only) Total cohort, low versus high, HR (95% CI): Low: 1.08 (0.84–1.40) High: 1 (reference) Premenopausal cohort, low versus high, HR (95% CI): Low: 1.67 (1.06–2.63) High: 1 (reference) Postmenopausal cohort, low versus high, HR (95% CI): Low: 0.93 (0.66–1.30) High: 1 (reference) Total cohort, quartiles, HR (95% CI): Q1: 1.05 (0.73–1.51) Q2: 0.99 (0.72–1.37) Q3: 1.07 (0.79–1.45) Q4: 1 (reference) Premenopausal cohort, quartiles, HR (95% CI): Q1: 1.21 (0.64–2.28) Q2: 0.99 (0.56–1.76) Q3: 0.84 (0.47–1.48) Q4: 1 (reference) Postmenopausal cohort, quartiles, HR (95% CI): Q1: 0.96 (0.58–1.56) Q2: 0.97 (0.62–1.49) Q3: 1.28 (0.86–1.89) Q4: 1 (reference) No association was observed in either stratum of baseline menopausal status when HDL was analyzed as a continuous variable (per 1 SD increment) Exclusion of the first 5 years of follow‐up did not appreciably change the observed associations
Lee et al. (2017)^60^	Prospective, cohort study	Korea	23,820	50–64 years	131	HDL‐C, mg/dl, low versus high (<40 mg/dl vs. ≥40 mg/dl)	Low HDL‐C, an individual component of MetS, was not associated with breast cancer risk Analyses adjusted for age and BMI; RR (95% CI): Low: 0.63 (0.28–1.45); *p* = 0.278 High: 1 (reference)
Lucht et al. (2019)^55^	Prospective, nested case–control study	USA	1912	Mean age at blood draw: 55.7–57.2 years (controls)	937 (975 controls)	Total cholesterol, mg/dl, tertiles: T1: 117.2–202.0 T2: 202.0–232.9 T3: 233.0–351.8	Among postmenopausal women, no association between total cholesterol and breast cancer risk was observed, irrespective of PMD (*p*_interaction_ 0.83) Model adjusted for BMI, fasting status, time of blood draw, age, alcohol consumption, race, family history of breast cancer, prior diagnosis of benign breast disease, age at menarche, BMI at age 18 years, menopause hormone therapy, and parity/age at first birth
			865		416 (449 controls)	HDL‐C, mg/dl, tertiles: T1: 10.4–52.9 T2: 52.9–67.0 T3: 67.1–125.9	Among postmenopausal women, no association between HDL‐C and breast cancer risk, irrespective of PMD (*p*_interaction_ 0.80) Model adjusted for BMI, fasting status, time of blood draw, age, alcohol consumption, race, family history of breast cancer, prior diagnosis of benign breast disease, age at menarche, BMI at age 18 years, menopause hormone therapy, and parity/age at first birth
Manjer et al. (2001)^64^	Prospective, cohort study	Sweden	9738	Mean (SD): 49.6 (7.8) years (overall) 42.8 (7.9) years (premenopausal) 54.1 (3.0) years (peri/postmenopausal)	326	Cholesterol, mmol/L, quartiles Premenopausal women: Q1: ≤ 4.61 Q2: 4.61–5.35 Q3: 5.35–6.16 Q4: >6.16 Peri/postmenopausal women: Q1: ≤ 5.56 Q2: 5.56–6.30 Q3: 6.30–6.90 Q4: >6.90	In premenopausal women, cholesterol did not affect the relative risk of breast cancer. In peri/postmenopausal women, the relative risk was increased over quartiles of cholesterol Model adjusted for age Premenopausal cohort: RR (95% CI): Q1: 1 (reference) Q2: 0.72 (0.42–1.21) Q3: 0.79 (0.46–1.36) Q4: 1.12 (0.64–1.97); *p*_trend_ 0.49 Peri/postmenopausal cohort: RR (95% CI): Q1: 1 (reference) Q2: 1.04 (0.66–1.63) Q3: 1.64 (1.05–2.56) Q4: 1.43 (0.91–2.25); *p*_trend_ 0.05
Melvin et al. (2012)^56^	Prospective, cohort study	Sweden	27,394	Mean (SD): 51.31 (11.58) years (cases) 46.68 (13.68) years (non‐cases)	6105	Total cholesterol, LDL‐C, and HDL‐C	No association was observed between levels of total cholesterol, LDL‐C, or HDL‐C with risk of breast cancer.
						Total cholesterol, mmol/L, quartiles: Q1: <4.80 Q2: 4.80–5.50 Q3: 5.50–6.30 Q4: ≥6.30	Model adjusted for age, glucose, parity, TGs, fasting status, and socioeconomic status, by quartiles, HR (95% CI): Q1: 1 (reference) Q2: 1.13 (1.05–1.22) Q3: 1.06 (0.98–1.14) Q4: 0.97 (0.89–1.05), *p*_trend_ 0.20
						LDL‐C, mmol/L, quartiles: Q1: <2.72 Q2: 2.72–3.37 Q3: 3.37–4.14 Q4: ≥4.14	Model adjusted for age, glucose, parity, TGs, fasting status, and socioeconomic status, by quartiles, HR (95% CI): Q1: 1 (reference) Q2: 1.10 (0.91–1.32) Q3: 1.00 (0.82–1.22) Q4: 0.92 (0.75–1.13), *p*_trend_ 0.29
						HDL‐C, mmol/L, quartiles: Q1: <1.45 Q2: 1.45–1.70 Q3: 1.70–1.98 Q4: ≥1.98	Model adjusted for age, glucose, parity, TGs, fasting status, and socioeconomic status, by quartiles, HR (95% CI): Q1: 1 (reference) Q2: 0.95 (0.79–1.15) Q3: 1.02 (0.84–1.25) Q4: 1.05 (0.86–1.29), *p*_trend_ 0.45
Pedersen et al. (2020)^47^	Prospective cohort study	Copenhagen, Denmark	Copenhagen General Population Study (*n* = 56,790)	≥20 years	1641	HDL‐C, mmol/L, cutoff points: <1.0 1.0–1.4 1.5–1.9 ≥2.0 HDL‐C, per 0.5 mmol/L or 19 mg/dl (1 SD) decrease	Low HDL‐C levels were associated with a minor increase in breast cancer risk Multivariate analysis adjusted for age, sex, BMI, smoking status, cumulative tobacco consumption, alcohol intake, leisure‐time physical activity, education, income, plasma TGs, lipid‐lowering therapy, C‐reactive protein, and baseline chronic disease (ischemic heart disease, chronic obstructive pulmonary disease, and diabetes) Cutoff points, HR (95% CI): <1.0: 1.15 (0.74–1.77) 1.0–1.4: 1.36 (1.11–1.66) 1.5–1.9: 1.20 (1.01–1.41) ≥2.0: 1 (reference), *p*_trend_ 0.008 Per 1 SD decrease, HR (95% CI): 1.12 (1.03–1.22), *p*_trend_ 0.007
Schairer et al. (2020)^62^	Retrospective, nested case–control study	USA	Kaiser Permanente Northern California (>4 million members)	25–95 years	*IBC subgroup* Cases: 247 Controls: 2470	HDL‐C, mg/dl, cutoff points: <50.00 50.00–64.99 ≥65.00 HDL‐C, mg/dl, categorical variable as continuous	Low HDL‐C levels increase IBC risk independently of the BMI association Analyses adjusted for age, service area, number of encounters, race, and mammographic screening Total IBC cohort HDL, cutoff points, OR (95% CI): <50.00: 2.6 (1.7–4.0) 50.00–64.99: 2.0 (1.3–3.0) ≥65.00: 1 (reference) Total IBC cohort HDL, categorical variable as continuous, OR (95% CI): 1.6 (1.3–1.9) ER+ IBC cohort HDL, cutoff points, OR (95% CI): <50.00: 2.9 (1.6–5.2) 50.00–64.99: 1.8 (1.0–3.1) ≥65.00: 1 (reference) ER+ IBC cohort HDL, categorical variable as continuous, OR (95% CI): 1.7 (1.3–2.2) ER‐ IBC cohort HDL, cutoff points, OR (95% CI): <50.00: 2.2 (1.1–4.5) 50.00–64.99: 2.2 (1.1–4.6) ≥65.00: 1 (reference) ER‐ IBC cohort HDL, categorical variable as continuous, OR (95% CI): 1.4 (1.0–1.9) *p* value for comparing ER+ to ER‐: 0.58
Strohmaier et al. (2013)^32^	Prospective cohort study	Norway, Austria, Sweden	288,057	Mean: 40.3–47.5 years	5228	Cholesterol, mmol/L, quintiles Cholesterol, per 1 mmol/L increment	Total serum cholesterol was inversely associated with breast cancer risk Models used age as the timescale, stratified by cohort, fasting status, and birth year categories, adjusted for baseline age, BMI, and smoking status Quintiles, HR (95% CI): Q1: 1 (reference) Q2: 0.93 (0.81–1.07) Q3: 0.91 (0.79–1.04) Q4: 0.84 (0.73–0.96) Q5: 0.70 (0.61–0.81); *p*_trend_ < 0.01 Per 1 mmol/L increment, HR (95% CI): 0.90 (0.86–0.93)

Reference	Mendelian randomization studies		
	Country/Region	Study name/cohort	Main conclusions/outcomes
Orho‐Melander et al. (2018)^49^	Sweden	Malmö Diet and Cancer Study (*n* = 26,904; mean [SD] age at baseline: 57.8 [7.6] years) Breast cancer cases: *n* = 1187 GWAS for lipids: Teslovich TM et al. Biological, clinical and population relevance of 95 loci for blood lipids^81^	LDL IV and HDL IV were not related to incident risk of breast cancer in logistic regression analysis or in multivariable pleiotropy‐adjusted analysis **Breast cancer** LDL IV, per SD increment: OR (95% CI): 1.05 (0.84–1.31) LDL IV multivariable, per SD increment: OR (95% CI): 1.05 (0.87–1.26) LDL serum, per SD increment: OR (95% CI): 0.89 (0.78–1.00) HDL IV, per SD increment: OR (95% CI): 1.25 (0.99–1.59) HDL IV multivariable, per SD increment: OR (95% CI): 1.11 (0.93–1.32) HDL serum, per SD increment: OR (95% CI): 1.07 (0.93–1.23)
Beeghly‐Fadiel et al. (2020)^65^	European ancestry	GWAS: Global Lipids Genetics Consortium Risk analysis: BCAC Cases: *n* = 101,424 Controls: *n* = 80,253 Mean age at diagnosis for cases or age at interview for controls: 55.8–56.6 years	Strong evidence that HDL may be associated with an increased risk of breast cancer, whereas LDL may not be related to breast cancer risk (all models adjusted for age, study site, or country, and principal components for European ancestry) One SD increase in genetically predicted HDL (~15 mg/dl) was associated with a 12% increased risk of breast cancer (OR [95% CI]: 1.12 [1.08–1.16]; *p* = 1.7 × 10^−9^) No association was found between genetically predicted LDL and breast cancer risk LDL, per 1 SD increase in genetically predicted LDL (~37 mg/dl): OR (95% CI): 1.00 (0.96–1.04); *p =* 0.88 No significant associations were found between genetically predicted total cholesterol and breast cancer risk Total cholesterol, per 1 SD increase in genetically predicted total cholesterol (~42 mg/dl): OR (95% CI): 1.05 (0.99–1.11); *p =* 0.11
Johnson et al. (2020)^66^	European ancestry	GWAS: Million Veteran Program (*n* = 215,551) Risk analysis: BCAC Cases: *n* = 122,977 Controls: *n* = 105,974	Genetically elevated plasma HDL and LDL levels appear to be associated with increased breast cancer risk Single‐trait MR for HDL, per 1 SD increase: OR (95% CI): 1.08 (1.04–1.13); *p* < 0.001 Multivariable MR (adjusted for effects of LDL, TGs, BMI, and age at menarche) for HDL per 1 SD increase supported the single‐trait observation for HDL: OR (95% CI): 1.06 (1.03–1.10); *p* = 4.93 × 10^−4^ Multivariable MR analysis for LDL per 1 SD increase showed a relationship between LDL and breast cancer risk: OR (95% CI): 1.03 (1.01–1.07); *p* = 0.02 No difference in the relationships for HDL or LDL were observed when stratified by breast tumor ER status
Nowak and Ärnlöv (2018)^67^	European ancestry	GWAS: Global Lipids Genetic Consortium (*n* ≤ 188,578) BCAC Cases: *n* = 61,282 Controls: *n* = 45,494	Genetically raised LDL was associated with higher risk of breast cancer: OR (95% CI) per SD: 1.09 (1.02–1.18); *p* = 0.020, and higher risk of ER+ breast cancer: OR (95% CI) per SD: 1.14 (1.05–1.24); *p* = 0.004, with no association with ER‐ breast cancer (*p* = 0.577) Genetically raised HDL had no nominally significant association with overall breast cancer risk: OR (95% CI) per SD: 1.07 (0.97–1.19); *p* = 0.171, or ER‐ breast cancer: OR (95% CI) per SD: 1.09 (0.91–1.30); *p* = 0.365, but appeared associated with higher risk of ER+ breast cancer: OR (95% CI) per SD: 1.13 (1.01–1.26); *p* = 0.037 LDL‐raising variants in PCSK9 were associated with increased breast cancer risk: OR (95% CI) per SD: 1.10 (1.02–1.19); *p* = 0.014, but not with ER+ (*p* = 0.099) or ER‐ (*p* = 0.089) breast cancer

Abbreviations: BMI, body mass index; CI, confidence interval; ER, estrogen receptor; GWAS, genome‐wide association study; HDL‐C, high‐density lipoprotein cholesterol; HR, hazard ratio; HRT, hormone replacement therapy; IBC, inflammatory breast cancer; IV, instrument variable; LDL‐C, low‐density lipoprotein cholesterol; MetS, metabolic syndrome; MR, Mendelian randomization; non‐HDL‐C, non‐high‐density lipoprotein cholesterol; OR, odds ratio; PMD, percent mammographic density; PR, progesterone receptor; RR, rate ratio; SD, standard deviation; SE, standard error; TG, triglyceride.

^a^ Age at baseline or recruitment.

The effect of total serum cholesterol levels on incident breast cancer risk varied among the studies identified. An analysis of the Women's Health Study cohort, including 15,602 women aged ≥45 years and without cardiovascular disease, use of hormone replacement therapy, or use of lipid‐lowering therapies at baseline, demonstrated no association between quartiles of total cholesterol (Q4: 235–474 mg/dl vs. Q1: 72–180 mg/dl: HR [95% CI] 0.98 [0.80–1.19]; *p*_trend_ 0.96) after model adjustment for age and treatment (aspirin, vitamin E, or β‐carotene) or per 1 SD increase (HR [95% CI]: 0.96 [0.89–1.03]) and breast cancer risk.^51^ Furthermore, in stratified analyses by age (<55 vs. ≥55 years) and BMI (<30 vs. ≥30), no association between the highest quartile compared to the lowest quartile of total cholesterol levels and breast cancer was observed.^51^ Similarly, an analysis within the prospective Nurses' Health Study reported no association between quintiles of total serum cholesterol levels and breast cancer risk in either pre‐ or postmenopausal women.^52^ Additionally, there was no association after excluding cases diagnosed in the first 2 years of follow‐up.^52^ A nested case–control study within the prospective de Epidémiologique auprès des Femmes de la Mutuelle Générale de l'Education Nationale (E3N) study also reported no association between tertiles of total serum cholesterol (T1: <4.87, T2: 4.87–5.55, T3: ≥5.56 mmol/L) and risk of breast cancer; furthermore, the risk was not modified by BMI, menopausal status, waist circumference, time between blood donation and diagnosis, or ER status of the tumor.^53^ No association between quartiles of total serum cholesterol levels (Q1: <4.80; Q2: 4.80–<5.50, Q3: 5.50–<6.30, Q4: ≥6.30 mmol/L) and risk of breast cancer was also reported following an analysis of the Swedish AMORIS database.^56^ In addition, when excluding those with follow‐up less than 3 years, no link was observed.^56^ In a prospective study of 24,208 postmenopausal women in the Women's Health Initiative, quartiles of total cholesterol showed no association with cancers of the breast even after accounting for possible sources of confounding and bias.^54^, ^55^ In a prospective, nested case–control study, Lucht et al. also demonstrated no association between tertiles of total cholesterol (T1: 117.2–202.0; T2: 202.0–232.9; T3: 233.0–351.8 mg/dl) and risk of breast cancer among postmenopausal women; a sensitivity analysis after excluding cases diagnosed within 12 months also showed null results.^54^, ^55^ A prospective study within the Malmö Preventive Project (*n* = 9738; 326 cases) reported that the relative risk of breast cancer had no significant trends over quartiles of total cholesterol levels in perimenopausal/postmenopausal women (Q4 vs. Q1, RR [95% CI]: 1.43 [0.91–2.25] *p*_trend_ 0.05); furthermore, total cholesterol did not affect breast cancer risk in premenopausal women (Q4 vs. Q1, RR [95% CI]: 1.12 [0.64–1.97]; *p*_trend_ 0.49).^64^

In comparison, a positive association between serum total cholesterol levels and risk of breast cancer was reported in three studies.^41^, ^58^, ^64^ An analysis among a large cohort (*n* = 94,555 female participants; 5380 cases) of the National Institute of Health‐American Association of Retired Persons (NIH‐AARP) Diet and Health Study reported a consistent positive association between elevated cholesterol (self‐reported) and overall risk of breast cancer (HR [95% CI]: 1.13 [1.04–1.23]), which was maintained in a normal BMI subgroup.^58^ An earlier analysis conducted within a large prospective study in Korea demonstrated a positive association between high (≥240 mg/dl) versus low (<160 mg/dl) total cholesterol levels and breast cancer in women (HR [95% CI]: 1.17 [1.03–1.33]); the observed association was strengthened following exclusion of the first 5 years of follow‐up (HR [95% CI]: 1.21 [1.04–1.41]).^41^ Of note, three studies reported an inverse association between quartiles or quintiles of total serum cholesterol levels and breast cancer.^32^, ^33^, ^59^ From an analysis of women from a Norwegian cohort (*n* = 38,823; 708 cases), an inverse association between total serum cholesterol was reported for those with cholesterol in the highest quartile (>6.82 mmol/L) compared with the lowest quartile (<5.24 mmol/L) and postmenopausal breast cancer (age‐adjusted RR [95% CI] 0.63 [0.48–0.82]; *p*_trend_ 0.005). However, it should be noted that no association was reported among overweight and obese women.^59^ A prospective analysis conducted among 7557 participants (141 cases) of the Supplémentation en Vitamines et Minéraux Antioxydants (SU.VI.MAX) study reported an inverse association between total cholesterol and breast cancer risk (per 1 mmol/L increment, HR [95% CI]: 0.83 [0.69–0.99]; *p*_trend_ 0.04).^33^ Likewise, an analysis of participants in the Metabolic syndrome and Cancer Project (Me‐Can) reported an inverse association between total cholesterol levels (by quintiles and per 1 mmol/L increment) and breast cancer incidence, with associations persisting for comparisons between the fifth to the first quintile when excluding the first year of follow‐up.^32^

LDL‐C levels were not associated with breast cancer in the five reported studies.^33^, ^51^, ^53^, ^54^, ^56^ This agrees with the associations between total cholesterol and breast cancer reported from these studies, as summarized above. The only exception was the analysis from participants among the SU.VI.MAX cohort which reported no association between derived LDL‐C levels (Planella's formula) and breast cancer (e.g., per 1 mmol/L increment, HR [95% CI]: 0.83 [0.66–1.05]; *p*_trend_ 0.1); interestingly, the same study reported that total cholesterol and HDL‐C were associated with breast cancer risk (results summarized above and in Table 3). The results were consistent when excluding cases diagnosed during the first 3 years of follow‐up.^33^

In total, 12 studies reported on the association between HDL‐C levels and breast cancer. A prospective study evaluating the association of breast cancer with lipid biomarkers in participants from the Women's Health Study reported no association between HDL‐C levels and breast cancer risk (by quartiles, Q4 vs. Q1, HR [95% CI]: 0.96 [0.78–1.19]; *p*_trend_ 0.72; per 1 SD [51.1 mg/dl] increase, HR [95% CI]: 0.96 [0.89–1.04]).^51^ A smaller prospective case–control study within the E3N population also demonstrated no association between HDL‐C levels and breast cancer risk overall, either when assessed per doubling of HDL‐C levels (OR [95% CI], 0.98 [0.58–1.64]) or by HDL‐C tertiles (T3 vs. T1, OR [95% CI], 0.98 [0.74–1.30]; *p* = 0.84); the results were not modified when assessed by menopausal status.^53^ Similarly, no association between breast cancer and HDL‐C levels was reported in cohort studies from Korea (assessed by HDL‐C < 40 vs. ≥40 mg/dl)^60^ and Sweden (assessed by HDL quartiles).^56^ Another study that reported no association between HDL‐C levels (assessed by HDL‐C tertiles) and breast cancer was among postmenopausal women who were enrolled in two large prospective cohorts in the USA.^55^ In contrast, five studies reported an inverse association between HDL‐C levels and breast cancer risk, including a prospective nested case–control study among postmenopausal women of the ORDET cohort (HDL‐C ≤ 55 vs. >55 mg/dl, RR [95% CI]: 1.60 [1.10–2.33]).^61^ A second nested case–control study among participants from the USA reported a negative association between low HDL‐C levels (assessed using cutoff points [<50.00, 50.00–64.99, and ≥65.00 mg/dl (reference)] or as a continuous variable) and the increased risk of inflammatory breast cancer (OR [95% CI]: for <50.00 mg/dl: 2.6 [1.7–4.0]; for 50.00–64.99 mg/dl: 2.0 [1.3–3.0]); no significant difference in results was observed when assessed by ER status of cases.^62^ An analysis among two Norwegian population‐based screening surveys reported an inverse association between quartiles of HDL‐C and postmenopausal breast cancer risk (combined cohort, Q4 vs. Q1, RR [95% CI]: 0.75 [0.58–0.97]; *p*_trend_ 0.02); no significant association was observed in the premenopausal cohort.^59^ Two further prospective studies among cohorts based in France and Denmark also reported an inverse association between HDL‐C levels (assessed by quartiles, per 1 SD [19 mg/dl] increment, and per one unit increment) and breast cancer risk.^33^, ^47^ Of note, the inverse association between HDL‐C and breast cancer risk in the French cohort was borderline non‐significant when assessed according to menopausal status (premenopausal: per one unit increment in HDL‐C, HR [95% CI]: 0.31 [0.10–1.00], *p*_trend_ 0.05; postmenopausal: per one unit increment in HDL‐C, HR [95% CI]: 0.53 [0.28–1.01], *p*_trend_ 0.05).^33^ Finally, two studies reported borderline inverse associations between HDL levels and risk of breast cancer. An analysis among postmenopausal women of the Women's Health Initiative cohort reported a borderline association between HDL quartiles and breast cancer risk (Q4 vs. Q1, HR [95% CI]: 0.83 [0.68–1.01]; *p*_trend_ 0.03), which strengthened when excluding users of hormone therapy (Q4 vs. Q1, HR [95% CI]: 0.75 [0.59–0.93]; *p*_trend_ 0.004).^54^ Although an analysis by Kucharska‐Newton and colleagues among participants of the Atherosclerosis Risk in Communities (ARIC) study found no association between HDL‐C levels and breast cancer risk for the overall cohort (<50 vs. ≥50 mg/dl, HR [95% CI]: 1.08 [0.84–1.40]; Q1 vs. Q4, HR [95% CI]: 1.05 [0.73–1.51]), they reported a nonsignificant signal for low HDL among premenopausal women as a marker for increased breast cancer risk (<50 vs. ≥50 mg/dl, HR [95% CI]: 1.67 [1.06–2.63]; Q1 vs. Q4, HR [95% CI]: 1.21 [0.64–2.28]).^63^

### 3.5 Breast cancer findings across Mendelian randomization studies

Four Mendelian randomization studies were identified that evaluated the association between circulating lipids and breast cancer (Table 3; Table S3).^49^, ^65^, ^66^, ^67^ All four studies had very large sample sizes with >1000 cases, used two‐sample Mendelian randomization designs, and combined SNPs to form genetic scores as the main predictor in the model. The number of SNPs included in the genetic scores for TGs, LDL‐C, and HDL‐C were 4, 44, and 28 for the Nowak et al. study^67^; 19, 30, and 32 for the Johnson et al. study^66^; 43, 57, and 74 for the Beeghly‐Fadiel et al. study^65^; and 26, 32, and 41 for the Orho‐Melander et al. study,^49^ respectively. Only one study examined the relationship between total cholesterol and breast cancer. No significant association was observed between genetically predicted increase in total cholesterol (~42 mg/dl) and breast cancer (OR [95% CI]: 1.05 [0.99–1.11]; *p* = 0.11).^65^

Four studies reported the association between genetically predicted increase in LDL‐C and breast cancer.^49^, ^65^, ^66^, ^67^ Two of these found a significant association between genetically predicted LDL‐C and breast cancer,^66^, ^67^ reporting ORs (95% CI) of 1.03 (1.01–1.07); *p* = 0.02 and 1.09 (1.02–1.18); *p* = 0.020, respectively. The other two studies found no association between genetically predicted LDL‐C and breast cancer.^49^, ^65^ Of the two studies examining relationships between LDL‐C and ER+ or ER‐ breast cancer,^66^, ^67^ one found an association. An association between genetically elevated LDL‐C (per SD increase) and a higher risk was observed for ER+ breast cancer (OR [95% CI]: 1.14 [1.05–1.24]; *p* = 0.004) but not for ER‐ breast cancer (*p* = 0.577).^67^ Furthermore, *PCSK9* genetic variants known to result in elevated LDL‐C levels were associated with increased breast cancer risk overall (per SD, OR [95% CI]: 1.10 [1.02–1.19]; *p* = 0.014).^67^ Notably, although Orho‐Melander et al. found no significant associations with the identified SNPs in the genetic scores for HDL‐C or LDL‐C, they found a significant decrease in the odds of breast cancer for each copy of HMGCR rs12916 allele, which mimics the effect of statins (OR [95% CI]: 0.89 [0.82–0.96] per LDL‐lowering T‐allele).^49^

Similar to the LDL‐C findings, half of the studies examining the relationship between HDL‐C and breast cancer observed a significant association.^65^, ^66^ In contrast, one study found no association with overall breast cancer,^49^ and one study found no association with overall breast cancer risk or ER‐ breast cancer.^67^ Only a modest effect was observed in the studies which found a significant association. These publications found between a 6% and 12% increased risk of breast cancer (OR [95% CI]: 1.06 [1.03–1.10]; *p* = 4.93 × 10^−4^ and 1.12 [1.08–1.16]; *p* = 1.7 × 10^−9^), respectively.^65^, ^66^ In the two studies examining ER status, one found no association^66^ and the other found an association with HDL‐C and a higher risk of ER+ breast cancer (per SD, OR [95% CI]: 1.13 [1.01–1.26]; *p* = 0.037).^67^

### 3.6 Endometrial cancer findings across longitudinal studies

The risk of endometrial cancer has risen substantially over the years, mainly due to increases in BMI.^68^ While there is a clear association between obesity and risk for endometrial cancer, it is still unknown whether lipoproteins, after controlling for obesity, have a major impact on the development of endometrial cancer. From a mechanistic perspective, high levels of adipose tissues increase estrogen by improving the efficiency of androstendione, leading to lower levels of peroxisome proliferator activated receptor alpha, which is the involved in the catabolism of lipoproteins.^69^, ^70^ To investigate the link between lipoproteins and endometrial cancer, we found five prospective cohort studies reporting on the association.^54^, ^71^, ^72^, ^73^, ^74^ A summary of the outcomes reported for endometrial cancer is presented in Table 4.

**TABLE 4:** Summary of outcomes reported for endometrial cancer

	Longitudinal studies						
Reference	Study design	Location	Cohort size	Age^a^	Number of cases	Exposure	Outcome
Bjørge et al. (2010)^71^	Prospective, cohort study	Austria, Norway, Sweden	287,320	Mean: 44 years	917	Cholesterol, mmol/L, quintiles, mean (SD): Q1: 4.2 (0.4) Q2: 4.9 (0.2) Q3: 5.5 (0.3) Q4: 6.1 (0.3) Q5: 7.3 (0.9)	No association between cholesterol and the risk of endometrial cancer and cholesterol Stratified by cohort and adjusted for year of birth and smoking, RR (95% CI): Q1: 1.00 (reference) Q2: 0.94 (0.74–1.20) Q3: 1.06 (0.84–1.33) Q4: 0.88 (0.70–1.11) Q5: 0.97 (0.78–1.22), *p*_trend_ 0.7 Stratified by cohort and adjusted for year of birth and smoking, and further adjusted for quintile of BMI, RR (95% CI): Q1: 1.00 (reference) Q2: 0.91 (0.71–1.16) Q3: 0.99 (0.79–1.25) Q4: 0.80 (0.64–1.01) Q5: 0.87 (0.70–1.10), *p*_trend_ 0.2
Cust et al. (2007)^72^	Prospective, nested case–control study	Denmark, France, Germany, Greece, Italy, The Netherlands, Spain, and the UK	135, 953	Mean (5th–95th percentile) age at blood collection: 56.9 (45.4–67.9) years (cases) 56.9 (45.0–68.0) years (controls)	284 (546 controls)	Total cholesterol, HDL‐C, and LDL‐C	HDL‐C was inversely Associated with risk of endometrial cancer; after adjustment for BMI, this association remained statistically significant. After further adjustment for sex steroid hormones and other obesity‐related hormones, the association between HDL‐C and endometrial cancer risk was no longer significant Total cholesterol and LDL‐C were not significantly associated with the overall risk of endometrial cancer Crude model: adjusted for matching factors only (study center, baseline menopausal status, age at blood collection, time of day of blood collection, fasting status, and phase of menstrual cycle [premenopausal women only]) BMI‐adjusted model: adjusted for matching factors (as noted above) and BMI Full adjustment model: adjusted for matching factors (as noted above) and BMI, C‐peptide, IGFBP‐1, IGFBP‐2, adiponectin, SHBG, free testosterone, and estrone
						Total cholesterol, mmol/L, quartiles: Q1: ≤ 4.36 Q2: 4.37–4.85 Q3: 4.86–5.40 Q4: ≥5.41	Crude model, RR (95% CI): Q1: 1 (reference) Q2: 0.82 (0.55–1.24) Q3: 0.65 (0.42–0.99) Q4: 0.92 (0.60–1.40), *p*_trend_ 0.45 BMI‐adjusted, RR (95% CI): Q1: 1 (reference) Q2: 0.80 (0.53–1.22) Q3: 0.66 (0.43–1.02) Q4: 0.90 (0.59–1.39), *p*_trend_ 0.45 Full adjustment, RR (95% CI): Q1: 1 (reference) Q2: 0.82 (0.44–1.51) Q3: 0.76 (0.36–1.61) Q4: 1.27 (0.49–3.29), *p*_trend_ 0.60
						HDL‐C, mmol/L, quartiles: Q1: ≤ 1.13 Q2: 1.14–1.35 Q3: 1.36–1.58 Q4: ≥1.59	Crude model, RR (95% CI): Q1: 1 (reference) Q2: 0.62 (0.41–0.93) Q3: 0.44 (0.28–0.68) Q4: 0.46 (0.30–0.72), *p*_trend_ 0.0003 BMI‐adjusted, RR (95% CI): Q1: 1 (reference) Q2: 0.69 (0.45–1.04) Q3: 0.50 (0.32–0.78) Q4: 0.61 (0.38–0.97), *p*_trend_ 0.02 Full adjustment, RR (95% CI): Q1: 1 (reference) Q2: 0.95 (0.56–1.60) Q3: 0.88 (0.46–1.68) Q4: 1.41 (0.54–3.68), *p*_trend_ 0.72
						LDL‐C, mmol/L, quartiles: Q1: ≤ 2.40 Q2: 2.40–2.88 Q3: 2.89–3.37 Q4: ≥3.38	Crude model, RR (95% CI): Q1: 1 (reference) Q2: 0.95 (0.63–1.43) Q3: 1.02 (0.68–1.54) Q4: 1.06 (0.70–1.61), *p*_trend_ 0.75 BMI‐adjusted, RR (95% CI): Q1: 1 (reference) Q2: 0.93 (0.61–1.41) Q3: 1.04 (0.68–1.57) Q4: 1.02 (0.66–1.56), *p*_trend_ 0.85 Full adjustment, RR (95% CI): Q1: 1 (reference) Q2: 1.22 (0.67–2.24) Q3: 1.61 (0.74–3.51) Q4: 2.14 (0.74–6.21), *p*_trend_ 0.16
Kabat et al. (2018)^54^	Prospective study	USA	24,208	Mean (SD): 64.1 (7.3) years (non‐cases) 63.6 (6.8) years (cases)	125	Total cholesterol, HDL‐C, and LDL‐C	In postmenopausal women, total cholesterol, HDL‐C, and LDL‐C were not associated with endometrial cancer risk Model adjusted for: age; smoking status; pack‐years of smoking; alcohol intake; BMI; physical activity; age at first birth; age at menarche; age at menopause; oral contraceptive use; hormone therapy; parous/nulliparous; education; ethnicity; and allocation to the observational study component or specific arm of clinical trials
						Total cholesterol, mg/dl, quartiles	HR (95% CI): Q1: 1 (reference) Q2: 0.77 (0.46–1.29) Q3: 0.92 (0.56–1.51) Q4: 0.88 (0.53–1.46), *p*_trend_ 0.79
						HDL‐C, mg/dl, quartiles	HR (95% CI): Q1: 1 (reference) Q2: 1.13 (0.71–1.82) Q3: 0.64 (0.37–1.12) Q4: 0.87 (0.51–1.50), *p*_trend_ 0.34
						LDL‐C, mg/dl, quartiles	HR (95% CI): Q1: 1 (reference) Q2: 0.85 (0.51–1.41) Q3: 0.75 (0.44–1.25) Q4: 0.87 (0.52–1.43), *p*_trend_ 0.53
Lindemann et al. (2009)^73^	Prospective study	Norway	31,473	Mean (SD): 48.8 (17.3) years (controls) 63.3 (10.96) years (cases)	100	Total cholesterol, HDL‐C, and LDL‐C	No association between total cholesterol, HDL‐C, and LDL‐C with endometrial cancer risk, either without or after adjustment for BMI
						Total cholesterol, mmol/L, quartiles: Q1: ≤ 5.0 Q2: 5.1–5.8 Q3: 5.9–6.8 Q4: ≥6.9	Model adjusted for age, other serum lipids and BMI, RR (95% CI): Q1: 1 (reference) Q2: 1.39 (0.52–3.67) Q3: 1.34 (0.42–4.29) Q4: 1.39 (0.35–5.47)
						LDL‐C, mmol/L, quartiles: Q1: ≤ 2.86 Q2: 2.87–3.59 Q3: 3.59–4.45 Q4 ≥4.46	Model adjusted for age, other serum lipids, and BMI, RR (95% CI): Q1: 1 (reference) Q2: 0.56 (0.23–1.33) Q3: 0.68 (0.25–1.85) Q4: 0.50 (0.15–1.63)
						HDL‐C, mmol/L, quartiles: Q1: ≤ 1.2 Q2: 1.3–1.5 Q3: 1.6–1.7 Q4: ≥1.8	Model adjusted for age, other serum lipids, and BMI, RR (95% CI): Q1: 1 (reference) Q2: 0.90 (0.54–1.52) Q3: 1.42 (0.78–2.64) Q4: 1.13 (0.57–2.26)
Seth et al. (2012)^74^	Prospective cohort study	Sweden	225,432	Mean (SD): 56.09 (10.06) years (cases) 46.52 (13.78) years (non‐cases)	1144	Total cholesterol, HDL‐C, and LDL‐C	An association between total cholesterol and risk of endometrial cancer risk was observed when analyzed by quartiles of total cholesterol, but not when using medical cutoff points for total cholesterol
						Total cholesterol, mmol/L, quartiles: Q1: <4.80 Q2: 4.80–5.50 Q3: 5.50–6.30 Q4: ≥6.30 Total cholesterol, mmol/L, <6.50 versus ≥6.50	Model adjusted for age, parity, glucose, TGs, fasting status, and socioeconomic status, HR (95% CI): Q1: 1 (reference) Q2: 1.28 (1.02–1.60) Q3: 1.28 (1.03–1.59) Q4: 1.15 (0.93–1.43), *p*_trend_ 0.731 Model adjusted for age, glucose, TGs, fasting status, and socioeconomic status, HR (95% CI): <6.50: 1 (reference) ≥6.50: 0.99 (0.87–1.12)
						HDL‐C, mmol/L, quartiles: Q1: <1.45 Q2: 1.45–1.70 Q3: 1.70–1.98 Q4: ≥1.98	Model adjusted for age, parity, glucose, TGs, fasting status, and socioeconomic status, HR (95% CI): Q1: 1 (reference) Q2: 1.03 (0.66–1.60) Q3: 0.72 (0.44–1.19) Q4: 0.97 (0.62–1.54), *p*_trend_ 0.645
						LDL‐C, mmol/L, quartiles: Q1: <2.70 Q2: 2.70–3.36 Q3: 3.36–4.13 Q4: ≥4.13	Model adjusted for age, parity, glucose, TGs, fasting status, and socioeconomic status, HR (95% CI): Q1: 1 (reference) Q2: 1.48 (0.85–2.56) Q3: 1.11 (0.64–1.94) Q4: 1.16 (0.67–2.00); *p*_trend_ 0.857

Reference	Mendelian Randomization Studies		
	Country/Region	Study name/cohort	Main conclusions/outcomes
Kho et al. (2021)^75^	European ancestry	GWAS: Global Lipids Genetic Consortium (*n* = 188,578) and Endometrial Cancer Association Consortium Cases: *n* = 12,906 Controls: *n* = 108,979	Genetically raised LDL was associated with lower risks of endometrial cancer of all histologies combined, and of endometrioid and non‐endometrioid subtypes All histologies, per 1 SD increase: OR (95% CI): 0.88 (0.83–0.93); *p =* 7.26 × 10^−6^ Endometrioid, per 1 SD increase: OR (95% CI): 0.89 (0.83–0.95); *p =* 5.37 × 10^−4^ Non‐endometrioid, per 1 SD increase: OR (95% CI): 0.76 (0.65–0.89); *p =* 6.18 × 10^−4^ Higher genetically predicted HDL was associated with increased risk of non‐endometrioid endometrial cancer All histologies, per 1 SD increase: OR (95% CI): 1.07 (1.00–1.14); *p = 0.037* Endometrioid, per 1 SD increase: OR (95% CI): 1.03 (0.95–1.11); *p* = 0.506 Non‐endometrioid, per 1 SD increase: OR (95% CI): 1.22 (1.01–1.47); *p = 0.036* This was n/s after adjusting for multiple comparisons

Abbreviations: BMI, body mass index; CI, confidence interval; HDL‐C, high‐density lipoprotein cholesterol; HR, hazard ratio; IGFBP, insulin‐like growth factor binding protein; LDL‐C, low‐density lipoprotein cholesterol; RR, relative risk; SD, standard deviation; SHBG, sex hormone‐binding globulin; TG, triglyceride.

^a^ Age at baseline or recruitment.

For the lipoproteins of interest, four of the five studies reported associations between the risk of endometrial cancer by total cholesterol, LDL‐C, and HDL‐C,^54^, ^72^, ^73^, ^74^ while one reported only on total cholesterol.^71^ Of the four studies reporting on LDL‐C, none found an association between LDL‐C and risk of endometrial cancer.^54^, ^72^, ^73^, ^74^ Additionally, while none of the five studies found a relationship between total cholesterol and the risk of endometrial cancer, Seth et al. reported a positive association between total cholesterol and risk of endometrial cancer for the second and third quartiles of total cholesterol compared with the first quartile (Q2 vs. Q1, HR [95% CI]: 1.28 [1.02–1.60]; Q3 vs. Q1, HR [95% CI]: 1.28 [1.03–1.59]; Q4 vs. Q1, HR [95% CI]: 1.15 [0.93–1.43]; *p*_trend_ 0.731). However, when the association was analyzed according to the National Cholesterol and Education Program thresholds for high cholesterol, <6.50 versus ≥6.50 mmol/L, instead of quartiles, the association no longer remained.^74^

Of the four studies that assessed the relationship between HDL‐C levels and incidence of endometrial cancer, only one reported an association.^72^ The authors reported an inverse association between quartiles of HDL‐C and risk of endometrial cancer (Q2 vs. Q1, HR [95% CI]: 0.62 [0.41–0.93]; Q3 vs. Q1, HR [95% CI]: 0.44 [0.28–0.68]; Q4 vs. Q1, HR [95% CI]: 0.46 [0.30–0.72]; *p*_trend_ 0.0003); however, after further adjustment for sex steroid hormones and other obesity‐related hormones, the observed association between HDL‐C and endometrial cancer was no longer significant (*p*_trend_ 0.09).^72^

### 3.7 Endometrial cancer findings across Mendelian randomization studies

One record reporting on a bidirectional, two‐sample Mendelian randomization analysis to investigate the relationship between circulating lipids (LDL‐C, HDL‐C, and TGs) and endometrial cancer risk was identified.^75^ Genetic variants associated with each of the lipid traits of interest were identified and assessed using data from two genome‐wide association studies (Global Lipids Genetics Consortium, *n* = 188,578^76^; and Endometrial Cancer Association Consortium, 12,906 cases and 108,979 controls).^77^ Genetically raised LDL‐C was reported to be associated with a lower risk of endometrial cancer, both overall and for endometrioid and non‐endometrioid subtypes (Table 4; Table S3). In contrast, a higher genetically predicted HDL‐C level was reported to be associated with an increased risk of non‐endometrioid endometrial cancer (*p* = 0.036); however, this finding was not significant after adjusting for multiple comparisons.

### 3.8 Ovarian cancer findings across longitudinal studies

Only two studies evaluated the relationship between circulating cholesterol levels and ovarian cancer.^54^, ^56^ A summary of the outcomes reported for ovarian cancer is presented in Table 5. An analysis among postmenopausal women from the Women's Health Initiative cohort (*n* = 24,208; 115 cases) reported no association between quartiles of total cholesterol, LDL‐C, and HDL‐C with ovarian cancer risk.^54^ Similarly, an analysis within the Swedish AMORIS database (*n* = 27,394; 808 cases) showed no association between circulating levels of total cholesterol, LDL‐C, and HDL‐C with risk of ovarian cancer (assessed by quartiles).^56^

**TABLE 5:** Summary of outcomes reported for ovarian cancer

Reference	Study design	Location	Cohort size	Age^a^	Number of cases	Exposure	Outcome
Kabat et al. (2018)^54^	Prospective study	USA	24,208	Mean (SD): 64.1 (7.3) years (non‐cases) 64.4 (6.7) years (cases)	115	Total cholesterol, HDL‐C, and LDL‐C	In postmenopausal women, total cholesterol, HDL‐C, and LDL‐C were not associated with ovarian cancer risk Model adjusted for: age; smoking status; pack‐years of smoking; alcohol intake; BMI; physical activity; age at first birth; age at menarche; age at menopause; oral contraceptive use; hormone therapy; parous/nulliparous; education; ethnicity; and allocation to the observational study component or specific arm of clinical trials
						Total cholesterol, mg/dl, quartiles	HR (95% CI): Q1: 1 (reference) Q2: 1.79 (1.02–3.14) Q3: 1.16 (0.62–2.14) Q4: 1.60 (0.89–2.86), *p*_trend_ 0.31
						HDL‐C, mg/dl, quartiles	HR (95% CI): Q1: 1 (reference) Q2: 1.03 (0.61–1.75) Q3: 0.95 (0.55–1.65) Q4: 0.61 (0.33–1.13), *p*_trend_ 0.09
						LDL‐C, mg/dl, quartiles	HR (95% CI): Q1: 1 (reference) Q2: 1.30 (0.74–2.28) Q3: 1.33 (0.76–2.33) Q4: 1.17 (0.65–2.09), *p*_trend_ 0.65
Melvin et al. (2012)^56^	Prospective, cohort study	Sweden	27,394	Mean (SD): 53.04 (11.64) years (cases) 46.68 (13.68) years (non‐cases)	808	Total cholesterol, LDL‐C, and HDL‐C	No association between total cholesterol, LDL‐C, or HDL‐C with risk of ovarian cancer was observed
						Total cholesterol, mmol/L, quartiles: Q1: <4.8 Q2: 4.80–5.50 Q3: 5.50–6.30 Q4: ≥6.30	Model adjusted for age, glucose, parity, TGs, fasting status, and socioeconomic status, HR (95% CI): Q1: 1 (reference) Q2: 1.12 (0.91–1.38) Q3: 1.11 (0.90–1.38) Q4: 1.07 (0.85–1.38), *p*_trend_ 0.68
						LDL‐C, mmol/L, quartiles: Q1: <2.72 Q2: 2.72–3.37 Q3: 3.37–4.14 Q4: ≥4.14	Model adjusted for age, glucose, parity, TGs, fasting status, and socioeconomic status, HR (95% CI): Q1: 1 (reference) Q2: 1.00 (0.56–1.77) Q3: 1.13 (0.64–1.98) Q4: 0.95 (0.53–1.71), *p*_trend_ 0.94
						HDL‐C, mmol/L, quartiles: Q1: <1.45 Q2: 1.45–1.70 Q3: 1.70–1.98 Q4: ≥1.98	Model adjusted for age, glucose, parity, TGs, fasting status, and socioeconomic status, HR (95% CI): Q1: 1 (reference) Q2: 0.97 (0.57–1.64) Q3: 0.91 (0.51–1.60) Q4: 0.95 (0.54–1.67); *p*_trend_ 0.81

Abbreviations: CI, confidence interval; HDL‐C, high‐density lipoprotein cholesterol; HR, hazard ratio; LDL‐C, low‐density lipoprotein cholesterol.

^a^ Age at baseline or recruitment.

There were no Mendelian randomization studies for ovarian cancer located in the literature.

## 4 CONCLUSIONS

The aim of our study was to summarize the association between serum cholesterol levels and incidence of hormonally driven cancers, with a focus on epidemiological studies with designs supporting the highest level of causal evidence. We identified 41 longitudinal or Mendelian randomization studies reporting on the association between circulating cholesterol levels and risk of prostate, breast, endometrial, or ovarian cancers. These results provide evidence for a complex association between cholesterol and different types of incident hormonally driven reproductive cancers. Even among the same types of cancer and serum cholesterol measures, often studies reported conflicting results and overall failed to convey evidence of an effect of plasma lipids.

Thus far, the evidence reviewed does not support an association between serum cholesterol variables (either total, HDL‐C, or LDL‐C levels) and incident ovarian cancer. Additionally, there were no observed associations between endometrial cancer and serum cholesterol variables among the longitudinal studies. However, the single Mendelian randomization study examining this relationship found that higher genetically predicted levels of HDL‐C and lower genetically predicted levels of LDL‐C were associated with incident endometrial cancer. The association with HDL‐C is likely spurious as the confidence interval for the comparison with HDL included 1 [estimate 1.07 (1.00–1.14)] and the *p*‐value was non‐significant after adjusting for multiple comparisons.^75^ As stated, this study also reported a protective effect of elevated LDL‐C levels. Despite this single finding, the preponderance of evidence suggests no association of serum cholesterol levels and endometrial cancer.

Similarly, the evidence reviewed does not support an association between cholesterol and prostate cancer. Most of the longitudinal studies and all the Mendelian randomization studies assessing the relationship between cholesterol and prostate cancer found no association. In the few studies reporting an association, the direction is inconsistent. Two publications reported an association between higher levels of total cholesterol and an increased risk of prostate cancer^41^, ^42^ and one reported lower levels of total cholesterol were associated with an increased risk of prostate cancer.^43^ The estimates from both Kitahara et al. and Kok et al. are derived from statistical models which are minimally adjusted; therefore, it is unclear whether either association would remain with additional adjustment. Finally, we cannot discount the results from the Heir et al. study due to the long duration of follow‐up and statistical analysis considering competing risks (i.e., death). As this is the only study reporting an inverse association, this relationship needs further exploration with similar statistical methods.

Finally, the association between cholesterol levels and breast cancer requires further research. Interestingly, our summary of longitudinal studies indicates a potential effect of lower HDL levels and increased breast cancer risk in some subgroups (i.e., post‐menopausal and obese). To examine these relationships often requires a large sample size and statistical evaluation of the interaction. These variables were not handled consistently across these studies. For example, menopausal status is often treated as a confounding variable^33^, ^51^, ^61^; however, there may be an interaction between serum cholesterol, menopausal status, and breast cancer development.^54^, ^58^, ^59^, ^63^, ^64^ In order to elucidate these potential interactions, future studies should be powered to evaluate potential interactions with menopausal status and obesity. Furthermore, only a modest effect of LDL and HDL on the risk of breast cancer was observed in the Mendelian randomization analyses, and potential pleiotropic effects cannot be ruled out. Therefore, these relationships require further exploration.

The limitations of this study include the inability to account for potential differences in risk factors related to cholesterol across geographic and ethnic populations. The data were not pooled for meta‐analysis due to the wide variety of populations, study designs, and serum cholesterol parameters included in the studies. Studies in this review were not formally assessed with a quality tool, but common weaknesses across included studies are insufficient sampling, short follow‐up time, and potential misclassification, such as pre‐clinical bias. In cases of pre‐clinical bias, undiagnosed cancer may already be affecting serum cholesterol as the cancer may utilize cholesterol for cell replication and therefore result in decreased serum levels. This could result in a mistaken association between low serum cholesterol and cancer diagnosis. The strengths of this analysis include the robustness of the review of studies and selection of those with the strongest epidemiologic study designs.

In summary, these results show no clear signal between serum cholesterol (total, HDL‐C, or LDL‐C) and incident, hormonally driven cancer. The apparent lack of a role for cholesterol in the risk of developing the hormonally driven cancers in the studies included in this review may be related to confounders, modifiers, or the design of such studies (limitations discussed above) that prevent clear patterns of association from emerging. While serum cholesterol levels do not necessarily reflect the status of intracellular cholesterol trafficking, these cells are reliant on circulating cholesterol for cancer development and growth. This need may be particularly elevated for malignant and fast‐growing tumors and for steroid hormone‐dependent cancers.

Thus, additional research into the role of cellular cholesterol is warranted. In addition, we would suggest conducting a rigorous life course epidemiologic study examining both the genetic and longitudinal association between serum lipids (in adolescence, young adulthood, and later adult life) and cancer risk. Such a study would help to elucidate biological and behavioral processes that operate in combination across an individual's life that influence cancer risk, accounting for multiple confounders such as diet, exercise, and use of lipid‐lowering therapy.

## AUTHOR CONTRIBUTIONS

**Dana J. Murdock:** Conceptualization (equal); formal analysis (equal); methodology (equal); writing – original draft (equal); writing – review and editing (equal). **Robert Sanchez:** Conceptualization (equal); formal analysis (equal); methodology (equal); writing – original draft (equal); writing – review and editing (equal). **Kusha A Mohammadi:** Conceptualization (equal); formal analysis (equal); methodology (equal); writing – review and editing (equal). **Sergio Fazio:** Conceptualization (equal); formal analysis (equal); methodology (equal); writing – review and editing (equal). **Gregory P. Geba:** Conceptualization (equal); formal analysis (equal); methodology (equal); writing – review and editing (equal).

## FUNDING INFORMATION

This analysis was funded by Regeneron Pharmaceuticals, Inc.

## CONFLICT OF INTEREST

All authors are employees of and stockholders in Regeneron Pharmaceuticals, Inc.

## Supporting information


Appendix S1

## Data Availability

Qualified researchers may request access to study documents (including the clinical study report, study protocol with any amendments, blank case report form, statistical analysis plan) that support the methods and findings reported in this manuscript. Individual anonymized participant data will be considered for sharing once the product and indication has been approved by major health authorities (e.g., FDA, EMA, and PMDA), if there is legal authority to share the data and there is not a reasonable likelihood of participant re‐identification. Submit requests to https://vivli.org/.
