# Supplementary material for: Serum cholesterol and the risk of developing hormonally driven cancers: A narrative review
Source: Cancer Med. 2022 Nov 29;12(6):6722–67. doi: 10.1002/cam4.5463 (PMC10067100; doi:10.1002/cam4.5463)
Supplement: Supplementary file 1 — Appendix S1 [file CAM4-12-6722-s001.docx]

Supplement

Supplemental FIG 1. PRISMA flow diagram of the search results


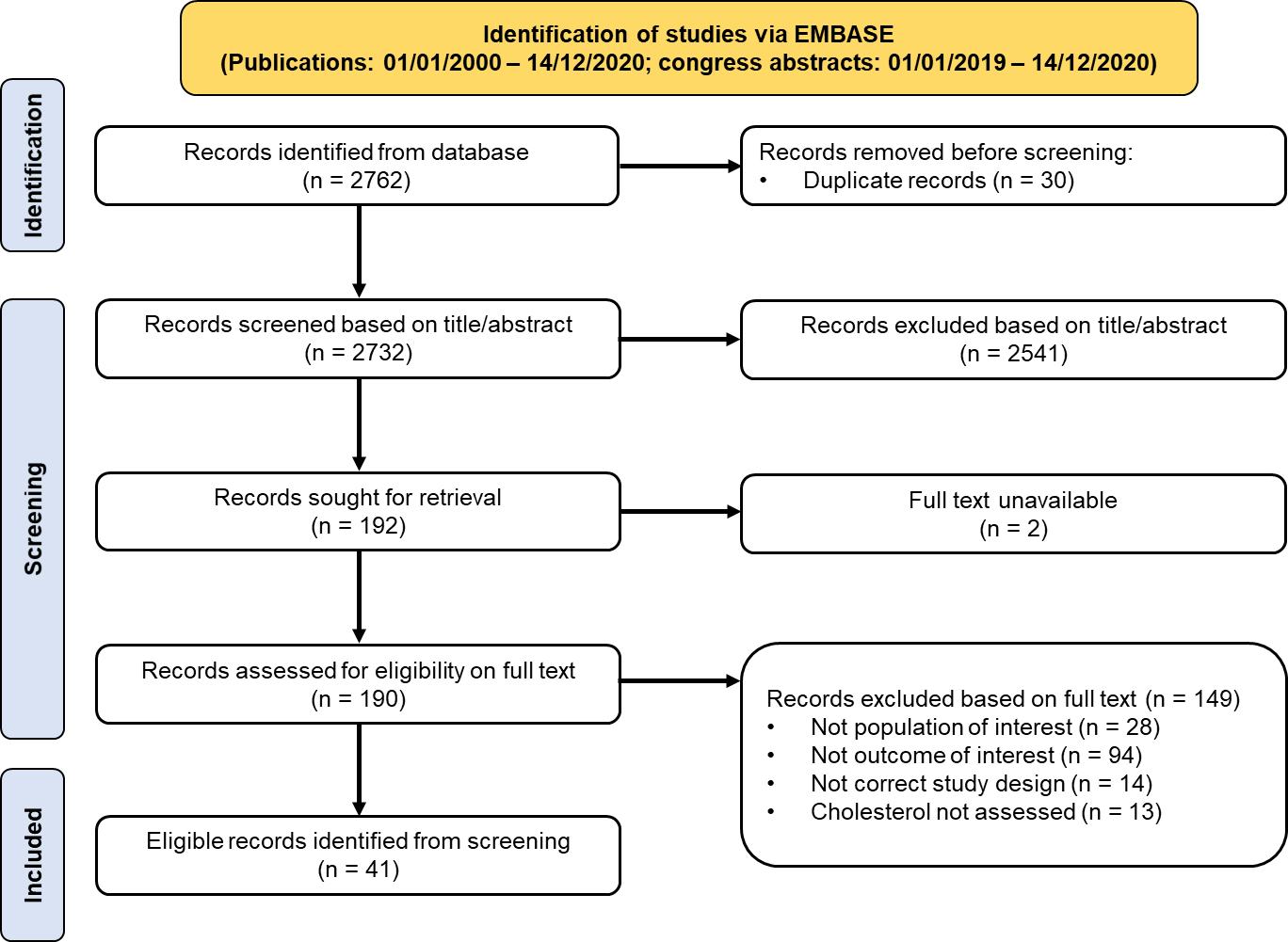


Supplemental TABLE 1. Search strategy for EMBASE (date of search: December 14, 2020)

| **Search** | **Query** | **Hits** |
| --- | --- | --- |
| **Cholesterol/high cholesterol** | |  |
| #1 | 'high density lipoprotein cholesterol'/exp OR 'low density lipoprotein cholesterol'/exp OR 'hypercholesterolemia'/exp OR 'lipoprotein'/exp OR 'dyslipidemia'/exp | 466,961 |
| #2 | cholesterol:ti,ab OR 'total cholesterol':ti,ab OR 'low-density lipoprotein':ti,ab OR 'high-density lipoprotein':ti,ab OR lipoprotein:ti,ab OR 'ldl-c':ti,ab OR ldl:ti,ab OR 'hdl-c':ti,ab OR hdl:ti,ab OR hypercholesterol*:ti,ab OR hyperlipid*:ti,ab OR dyslipid*:ti,ab OR dyslipoprotein*:ti,ab OR 'lipid profile*':ti,ab OR 'lipid component*':ti,ab OR 'blood lipid*':ti,ab OR 'plasma lipid*':ti,ab OR 'serum lipid*':ti,ab OR 'plasma lipoprotein*':ti,ab OR 'blood lipoprotein*':ti,ab OR 'serum lipoprotein*':ti,ab | 546,041 |
| #3 | #1 OR #2 | 725,734 |
| **Outcomes** | |  |
| #4 | 'prostate cancer'/exp OR 'ovary cancer'/exp OR 'ovary carcinoma'/exp OR 'breast cancer'/exp OR 'breast carcinoma'/exp OR 'endometrium cancer'/exp OR 'uterus cancer'/exp OR 'uterus carcinoma'/exp | 906,900 |
| #5 | 'prostate cancer':ti,ab OR 'breast cancer':ti,ab OR 'endometrial cancer':ti,ab OR 'ovarian cancer':ti,ab OR 'uterine cancer':ti,ab | 671,401 |
| #6 | breast*:ti,ab OR mammary:ti,ab OR endometri*:ti,ab OR ovarian:ti,ab OR ovary:ti,ab OR ovaries:ti,ab OR uterine:ti,ab OR uterus:ti,ab OR prostat*:ti,ab | 1,511,788 |
| #7 | tumor*:ti,ab OR tumour*:ti,ab OR cancer*:ti,ab OR malignancy:ti,ab OR malignancies:ti,ab OR neoplas*:ti,ab OR carcinoma*:ti,ab | 4,472,343 |
| #8 | #6 AND #7 | 951,860 |
| #9 | #4 OR #5 OR #8 | 1,169,180 |
| **Included study types** | |  |
| #10 | 'cohort analysis'/exp OR 'prospective study'/exp | 1,168,218 |
| #11 | (prospective*:ti,ab OR cohort*:ti,ab OR 'case-cohort':ti,ab OR 'nested case-control':ti,ab OR nested:ti,ab OR 'population-based':ti,ab OR 'post hoc' OR 'post-hoc') AND (study:ti,ab OR studies:ti,ab OR design*:ti,ab OR longitudinal:ti,ab OR registry:ti,ab OR epidemiol*:ti,ab OR 'follow-up':ti,ab OR 'follow up':ti,ab OR followed:ti,ab OR observation*:ti,ab OR analysis:ti,ab OR analyses:ti,ab) | 1,966,245 |
| #12 | 'systematic review'/exp OR 'systematic review topic'/exp OR [systematic review]/lim OR 'meta analysis'/exp OR 'meta analysis topic'/exp OR [meta analysis]/lim | 419,913 |
| #13 | (systematic AND (research OR review OR search OR overview)) OR (synthes* AND (literature OR research OR studies OR study OR data)) OR ((review OR research) AND (systematic* OR methodologic* OR quantitative OR effective*)) OR ('systematic review' OR 'systematic literature review' OR 'meta-analysis' OR 'meta-analyses' OR 'meta analysis' OR 'meta analyses') OR ('Mendelian randomization' OR 'Mendelian randomisation') | 3,318,927 |
| #14 | #10 OR #11 OR #12 OR #13 | 5,281,239 |
| **Unwanted study types** | |  |
| #15 | 'cross-sectional study'/exp | 380,145 |
| #16 | [editorial]/lim OR [erratum]/lim OR [letter]/lim OR 'case report':ti,ab OR 'case-report':ti,ab OR 'comment':ti,ab | 2,567,462 |
| #17 | 'chart review' OR 'random allocation' OR 'randomly assigned' OR 'randomly allocated' OR 'randomly divided' OR 'random order' | 352,179 |
| #18 | 'cell culture technique'/exp OR 'primary cell culture'/exp | 129,021 |
| #19 | 'animal model'/exp | 1,436,146 |
| #20 | #15 OR #16 OR #17 OR #18 OR #19 | 4,790,140 |
| **Cholesterol + outcomes + studies** | |  |
| #21 | #3 AND #9 AND #14 NOT #20 | 4,084 |
| **Filters (language, year, and congresses)** | |  |
| #22 | #3 AND #9 AND #14 NOT #20 AND [english]/lim | 3,996 |
| #23 | #3 AND #9 AND #14 NOT #20 AND [english]/lim AND [2000-2021]/py | 3,736 |
| #24 | #3 AND #9 AND #14 NOT #20 AND [english]/lim AND [2000-2021]/py AND [conference abstract]/lim | 999 |
| **Publications without congress abstracts** | |  |
| #25 | #23 NOT #24 | 2,737 |
| **Congress abstracts, 2 years** | |  |
| #26 | #24 AND [2019-2021]/py | 140 |
| **Selected congress abstracts, 2 years** | |  |
| #27 | #26 AND ('2019 annual meeting of the american society of clinical oncology, asco 2019':nc OR '2020 annual meeting of the american society of clinical oncology, asco 2020':nc OR '44th congress of european society for medical oncology, esmo 2019':nc OR '44th esmo congress':nc OR '5th european society for medical oncology asia congress, esmo 2019':nc OR 'american heart association scientific sessions, aha 2019':nc OR 'esmo virtual congress 2020':nc OR 'european society of cardiology congress, esc 2019':nc) | 25 |
| **Full-text publications, year 2000 – till date and congress abstracts, 2019 – till date** | |  |
| #28 | #25 OR #27 | 2,762 |

Supplemental TABLE 2. Summary of included longitudinal studies

| **Reference** | **Cancer type** | **Study design** | **Country** | **Study name/cohort** | **Enrolment time period** | **Follow-up** | **Categorization of cholesterol exposure** |
| --- | --- | --- | --- | --- | --- | --- | --- |
| Agnoli et al, 2010^1^ | Breast | Prospective, nested case-control study | Italy | ORDET, post-menopausal subgroup (n = 3,966) | 1987–1992 | Median, 13.5 years | HDL ≤ 55 mg/dL |
| Bjørge et al, 2010^2^ | Endometrial | Prospective, cohort study | Austria, Norway, Sweden | Me-Can (n = 287,320) | 1974–2005 | Average, 10 years | Cholesterol, by quintiles |
| Chandler et al, 2016^3^ | Breast | Prospective, cohort study | USA | Women’s Health Study (n = 15,602) | 1992–2004 | Median, 19 years | Total cholesterol, LDL-C, and HDL-C by quartiles  Total cholesterol, LDL-C, and HDL-C per 1 SD increase |
| Cust et al, 2007^4^ | Endometrial | Prospective, nested case-control study | Denmark, France, Germany, Greece, Italy, The Netherlands, Spain, and the UK | EPIC (n = 284 cases; n = 546 controls) | 1992–1998 | Not stated | Total cholesterol, LDL-C, and HDL-C by quartiles |
| Dibaba et al, 2018^5^ | Breast | Prospective, cohort study | USA | NIH-AARP Diet and Health Study (n = 94,555) | 1995–1996 | Mean, 14 years | Cholesterol, elevated (self-reported) |
| Eliassen et al, 2005^6^ | Breast | Prospective, cohort study | USA | Nurses’ Health Study (n = 71,921) | 1976 | 6–12 years; 665,743 person-years | Total cholesterol, self-reported |
| Furberg et al, 2004^7^ | Breast | Population-based screening survey | Norway | Norwegian National Health Screening Service (n = 38,823) | 1977–1983 and 1985–1987 | Median, 17.2 years | Total cholesterol and HDL-C by quartiles |
| Grundmark et al, 2010^8^ | Prostate | Prospective, longitudinal study | Sweden | ULSAM (n = 2,322) | 1970–1974 | 34 years | HDL-C |
| Häggström et al, 2012^9^ | Prostate | Prospective, cohort study | Austria, Norway, Sweden | Me-Can (n = 289,866) |  | Mean, 12 years | Cholesterol, quintiles |
| Håheim et al, 2006^10^ | Prostate | Prospective, cohort study | Norway | Oslo Study (n = 15,933) | 1972–1973 | 27 years | Total cholesterol, upper quartile |
| Heir et al, 2016^11^ | Prostate | Prospective, cohort study | Norway | Oslo Ischemia Study (n = 1,997) | 1972–1975 | 40 years | Total cholesterol, quartiles |
| His et al, 2017^12^ | Breast | Prospective, nested case-control study | France | E3N (n = 583 cases; n = 1,043 controls) | 1990 | NR | Total cholesterol, LDL-C, and HDL-C |
| His et al, 2014^13^ | Breast and prostate | Prospective study of RCT cohort | France | SU.VI.MAX (n = 7,557) | 1994–1995 | Mean, 11.5 years | Total cholesterol, LDL-C, and HDL-C by quartiles and by one SI unit increment |
| Kabat et al, 2018^14^ | Breast, endometrial, and ovarian | Prospective study | USA | Women’s Health Initiative (n = 24,208) | 1993–1998 | NR | Total cholesterol, LDL-C and HDL-C by quartiles |
| Kitahara et al, 2011^15^ | Breast and prostate | Prospective, cohort study | Korea | National Health Insurance Corporation (n = 1,189,719) | 1992–1995 | 14 years | Total cholesterol |
| Kok et al, 2011^16^ | Prostate | Prospective, population-based cohort study | The Netherlands | Nijmegan Biomedical Study (n = 2,118) | 2001–2003 | Median, 79.5 months | Total cholesterol, LDL-C and HDL-C |
| Kucharska-Newton et al, 2008^17^ | Breast | Prospective study | USA | ARIC (n = 7,575) | 1987–1989 | 13 years | HDL-C <50 mg/dL |
| Lee et al, 2017^18^ | Breast | Prospective, cohort study | Korea | NHIS-NSC (n = 23,820) | 2008–2009 | 5 years | HDL-C <40 mg/dL |
| Lindemann et al, 2009^19^ | Endometrial | Prospective study | Norway | HUNT II (n = 31,473) | 1995–1997 | 9 years | Total cholesterol, LDL-C, and HDL-C by quartiles |
| Lucht et al, 2019^20^ | Breast | Prospective, nested case-control study | USA | Nurses’ Health Study and Nurses’ Health Study II | 1976 and 1989 | NR | Total cholesterol, HDL-C, by tertiles |
| Manjer et al, 2001^21^ | Breast | Prospective, cohort study | Sweden | Malmö Preventive Project (n = 9,738) | 1977–1992 | 13.1 years | Cholesterol, by quartiles |
| Martin et al, 2009^22^ | Prostate | Prospective, cohort study | Norway | HUNT II (n = 29,364) | 1995–1997 | Mean, 9.3 years | Total cholesterol, HDL-C |
| Melvin et al, 2012^23^ | Breast and ovarian | Prospective, cohort study | Sweden | AMORIS (n = 27,394) | NR | Mean, 8 years | Total cholesterol, LDL-C, and HDL-C |
| Mondul et al, 2010^24^ | Prostate | Prospective study | USA | CLUE II (n = 6,816) | 1989 | Mean, 11.9 years | Cholesterol |
| Monroy-Iglesias et al, 2021^25^ | Prostate | Prospective cohort study | UK | UK Biobank (n = 220,622) | 2006–2010 | Median, 6.9 years | HDL-C |
| Pedersen et al, 2020^26^ | Breast and prostate | Prospective cohort study | Denmark | Copenhagen General Population Study (n = 107,341)  Copenhagen City Heart Study (n = 9,387) | 2003–2015  1991–1994 | 13 years  25 years | HDL-C |
| Platz et al, 2008^27^ | Prostate | Prospective, nested case-control study | USA | Health Professionals Follow-up Study (n = 18,018) | 1986 | NR | Cholesterol |
| Platz et al, 2009^28^ | Prostate | Prospective cohort study | USA | PCPT, placebo arm only (n = 5,586) | 1993–1996 | NR | Cholesterol |
| Schairer et al, 2020^29^ | Breast (inflammatory) | Retrospective, nested case-control study | USA | Kaiser Permanente Northern California | 2005–2017 | NR | HDL-C |
| Seth et al, 2012^30^ | Endometrial | Prospective cohort study | Sweden | AMORIS (n = 225,432) | 1985–1996 | Mean, 12.16 years | Total cholesterol, HDL-C, and LDL-C |
| Shafique et al, 2012^31^ | Prostate | Prospective cohort study | UK | Midspan (n = 12,926) | 1970–1976 | Median, 24 years | Cholesterol, quintiles |
| Strohmaier et al, 2013^32^ | Breast and prostate | Prospective cohort study | Norway, Austria, Sweden | Me-Can (males, n = 289,273; females, n = 288,057) | 1972–2005 | Mean, 11.7 years | Total cholesterol |
| Van Hemelrijck et al, 2011^33^ | Prostate | Prospective cohort study | Sweden | AMORIS (n = 200,660) | 1985–1996 | Mean, 6.96–8.27 years | Total cholesterol |
| Van Hemelrijck et al, 2011^34^ | Prostate | Prospective cohort study | Sweden | AMORIS (n = 69,735) | 1985–1996 | Mean, 11.5 years | Total cholesterol, HDL-C, and LDL-C |

AMORIS, Apolipoprotein MOrtality RISk; ARIC, Atherosclerosis Risk in Communities Study; E3N, Etude Epidémiologique auprès des Femmes de la Mutuelle Générale de l’Education Nationale; EPIC, European Prospective Investigation into Cancer and Nutrition; HDL-C, high-density lipoprotein cholesterol; HUNT II, second Nord Trøndelag Health Study; LDL-C, low-density lipoprotein cholesterol; Me-Can; Metabolic syndrome and Cancer project; NHIS-NSC, National Health Insurance Service-National Sample Cohort; NIH-AARP, National Institute of Health-American Association of Retired Persons; PCPT, Prostate Cancer Prevention Trial; SD, standard deviation; SU.VI.MAX, Supplémentation en VItamines et Minéraux Anti-oXydants; ULSAM, Uppsala Longitudinal Study of Adult Men.

Supplemental TABLE 3. Summary of included Mendelian randomization studies and outcomes

| **Reference** | **Cancer type** | **Country/Region** | **Study name/cohort** |
| --- | --- | --- | --- |
| Adams et al, 2019^35^ | Prostate | UK | GWAS:  Kettunen et al 2016^36^ and PRACTICAL consortium^37^  Risk analysis: ProtecT trial Cases: n = 2,291 Controls: n = 2,661 |
| Bull et al, 2016^38^ | Prostate | Australia, Bulgaria, Denmark, Europe, Finland, Germany, Poland, Portugal, Sweden, the USA, and the UK | GWAS: Do et al 2013^39^; Teslovich et al 2010^40^; Global Lipids Genetics Consortium^41^; Isaacs et al 2013^42^ Risk analysis: PRACTICAL consortium  Cases: n = 22,249 Controls: n = 22,133 |
| Orho-Melander et al, 2018^43^ | Breast and prostate | Sweden | Malmö Diet and Cancer Study (n = 26,904) Breast cancer cases: n = 1,187 Prostate cancer cases: n = 1,322  GWAS for lipids: Teslovich TM et al. Biological, clinical and population relevance of 95 loci for blood lipids.^40^ |
| Beeghly-Fadiel et al, 2020^44^ | Breast |  | GWAS: Global Lipids Genetics Consortium  Risk analysis: BCAC Cases: n = 101,424 Controls: n = 80,253 |
| Johnson et al, 2020^45^ | Breast |  | GWAS: Million Veteran Program (n = 215,551)  Risk analysis: BCAC Cases: n = 122,977 Controls: n = 105,974 |
| Nowak and Ärnlöv, 2018^46^ | Breast | Europe | GWAS: Global Lipids Genetic Consortium (n = 188,578)  BCAC Cases: n = 61,282 Controls: n = 45,494 |
| Kho et al, 2021^47^ | Endometrial | Europe | GWAS: Global Lipids Genetic Consortium (n = 188,578) and Endometrial Cancer Association Consortium Cases: n = 12,906 Controls:  n = 108,979 |

BCAC, Breast Cancer Association Consortium; GWAS, genome-wide association study.

# References

1. Agnoli C, Berrino F, Abagnato CA, Muti P, Panico S, Crosignani P, Krogh V. Metabolic syndrome and postmenopausal breast cancer in the ORDET cohort: a nested case-control study. *Nutr Metab Cardiovasc Dis.* 2010;20:41-48.

2. Bjorge T, Stocks T, Lukanova A, Tretli S, Selmer R, Manjer J, Rapp K, Ulmer H, Almquist M, Concin H, Hallmans G, Jonsson H, Stattin P, Engeland A. Metabolic syndrome and endometrial carcinoma. *Am J Epidemiol.* 2010;171:892-902.

3. Chandler PD, Song Y, Lin J, Zhang S, Sesso HD, Mora S, Giovannucci EL, Rexrode KE, Moorthy MV, Li C, Ridker PM, Lee IM, Manson JE, Buring JE, Wang L. Lipid biomarkers and long-term risk of cancer in the Women's Health Study. *Am J Clin Nutr.* 2016;103:1397-1407.

4. Cust AE, Kaaks R, Friedenreich C, Bonnet F, Laville M, Tjonneland A, Olsen A, Overvad K, Jakobsen MU, Chajes V, Clavel-Chapelon F, Boutron-Ruault MC, Linseisen J, Lukanova A, Boeing H, Pischon T, Trichopoulou A, Christina B, Trichopoulos D, Palli D, Berrino F, Panico S, Tumino R, Sacerdote C, Gram IT, Lund E, Quiros JR, Travier N, Martinez-Garcia C, Larranaga N, Chirlaque MD, Ardanaz E, Berglund G, Lundin E, Bueno-de-Mesquita HB, van Duijnhoven FJ, Peeters PH, Bingham S, Khaw KT, Allen N, Key T, Ferrari P, Rinaldi S, Slimani N, Riboli E. Metabolic syndrome, plasma lipid, lipoprotein and glucose levels, and endometrial cancer risk in the European Prospective Investigation into Cancer and Nutrition (EPIC). *Endocr Relat Cancer.* 2007;14:755-767.

5. Dibaba DT, Braithwaite D, Akinyemiju T. Metabolic Syndrome and the Risk of Breast Cancer and Subtypes by Race, Menopause and BMI. *Cancers (Basel).* 2018;10:299.

6. Eliassen AH, Colditz GA, Rosner B, Willett WC, Hankinson SE. Serum lipids, lipid-lowering drugs, and the risk of breast cancer. *Arch Intern Med.* 2005;165:2264-2271.

7. Furberg AS, Veierod MB, Wilsgaard T, Bernstein L, Thune I. Serum high-density lipoprotein cholesterol, metabolic profile, and breast cancer risk. *J Natl Cancer Inst.* 2004;96:1152-1160.

8. Grundmark B, Garmo H, Loda M, Busch C, Holmberg L, Zethelius B. The metabolic syndrome and the risk of prostate cancer under competing risks of death from other causes. *Cancer Epidemiol Biomarkers Prev.* 2010;19:2088-2096.

9. Haggstrom C, Stocks T, Ulmert D, Bjorge T, Ulmer H, Hallmans G, Manjer J, Engeland A, Nagel G, Almqvist M, Selmer R, Concin H, Tretli S, Jonsson H, Stattin P. Prospective study on metabolic factors and risk of prostate cancer. *Cancer.* 2012;118:6199-6206.

10. Lund Haheim L, Wisloff TF, Holme I, Nafstad P. Metabolic syndrome predicts prostate cancer in a cohort of middle-aged Norwegian men followed for 27 years. *Am J Epidemiol.* 2006;164:769-774.

11. Heir T, Falk RS, Robsahm TE, Sandvik L, Erikssen J, Tretli S. Cholesterol and prostate cancer risk: a long-term prospective cohort study. *BMC Cancer.* 2016;16:643.

12. His M, Dartois L, Fagherazzi G, Boutten A, Dupre T, Mesrine S, Boutron-Ruault MC, Clavel-Chapelon F, Dossus L. Associations between serum lipids and breast cancer incidence and survival in the E3N prospective cohort study. *Cancer Causes Control.* 2017;28:77-88.

13. His M, Zelek L, Deschasaux M, Pouchieu C, Kesse-Guyot E, Hercberg S, Galan P, Latino-Martel P, Blacher J, Touvier M. Prospective associations between serum biomarkers of lipid metabolism and overall, breast and prostate cancer risk. *Eur J Epidemiol.* 2014;29:119-132.

14. Kabat GC, Kim MY, Chlebowski RT, Vitolins MZ, Wassertheil-Smoller S, Rohan TE. Serum lipids and risk of obesity-related cancers in postmenopausal women. *Cancer Causes Control.* 2018;29:13-24.

15. Kitahara CM, Berrington de Gonzalez A, Freedman ND, Huxley R, Mok Y, Jee SH, Samet JM. Total cholesterol and cancer risk in a large prospective study in Korea. *J Clin Oncol.* 2011;29:1592-1598.

16. Kok DE, van Roermund JG, Aben KK, den Heijer M, Swinkels DW, Kampman E, Kiemeney LA. Blood lipid levels and prostate cancer risk; a cohort study. *Prostate Cancer Prostatic Dis.* 2011;14:340-345.

17. Kucharska-Newton AM, Rosamond WD, Mink PJ, Alberg AJ, Shahar E, Folsom AR. HDL-cholesterol and incidence of breast cancer in the ARIC cohort study. *Ann Epidemiol.* 2008;18:671-677.

18. Lee JA, Yoo JE, Park HS. Metabolic syndrome and incidence of breast cancer in middle-aged Korean women: a nationwide cohort study. *Breast Cancer Res Treat.* 2017;162:389-393.

19. Lindemann K, Vatten LJ, Ellstrom-Engh M, Eskild A. Serum lipids and endometrial cancer risk: results from the HUNT-II study. *Int J Cancer.* 2009;124:2938-2941.

20. Lucht SA, Eliassen AH, Bertrand KA, Ahern TP, Borgquist S, Rosner B, Hankinson SE, Tamimi RM. Circulating lipids, mammographic density, and risk of breast cancer in the Nurses' Health Study and Nurses' Health Study II. *Cancer Causes Control.* 2019;30:943-953.

21. Manjer J, Kaaks R, Riboli E, Berglund G. Risk of breast cancer in relation to anthropometry, blood pressure, blood lipids and glucose metabolism: a prospective study within the Malmo Preventive Project. *Eur J Cancer Prev.* 2001;10:33-42.

22. Martin RM, Vatten L, Gunnell D, Romundstad P, Nilsen TI. Components of the metabolic syndrome and risk of prostate cancer: the HUNT 2 cohort, Norway. *Cancer Causes Control.* 2009;20:1181-1192.

23. Melvin JC, Seth D, Holmberg L, Garmo H, Hammar N, Jungner I, Walldius G, Lambe M, Wigertz A, Van Hemelrijck M. Lipid profiles and risk of breast and ovarian cancer in the Swedish AMORIS study. *Cancer Epidemiol Biomarkers Prev.* 2012;21:1381-1384.

24. Mondul AM, Clipp SL, Helzlsouer KJ, Platz EA. Association between plasma total cholesterol concentration and incident prostate cancer in the CLUE II cohort. *Cancer Causes Control.* 2010;21:61-68.

25. Monroy-Iglesias MJ, Russell B, Crawley D, Allen NE, Travis RC, Perez-Cornago A, Van Hemelrijck M, Beckmann K. Metabolic syndrome biomarkers and prostate cancer risk in the UK Biobank. *Int J Cancer.* 2021;148:825-834.

26. Pedersen KM, Colak Y, Bojesen SE, Nordestgaard BG. Low high-density lipoprotein and increased risk of several cancers: 2 population-based cohort studies including 116,728 individuals. *J Hematol Oncol.* 2020;13:129.

27. Platz EA, Clinton SK, Giovannucci E. Association between plasma cholesterol and prostate cancer in the PSA era. *Int J Cancer.* 2008;123:1693-1698.

28. Platz EA, Till C, Goodman PJ, Parnes HL, Figg WD, Albanes D, Neuhouser ML, Klein EA, Thompson IM, Jr., Kristal AR. Men with low serum cholesterol have a lower risk of high-grade prostate cancer in the placebo arm of the prostate cancer prevention trial. *Cancer Epidemiol Biomarkers Prev.* 2009;18:2807-2813.

29. Schairer C, Laurent CA, Moy LM, Gierach GL, Caporaso NE, Pfeiffer RM, Kushi LH. Obesity and related conditions and risk of inflammatory breast cancer: a nested case-control study. *Breast Cancer Res Treat.* 2020;183:467-478.

30. Seth D, Garmo H, Wigertz A, Holmberg L, Hammar N, Jungner I, Lambe M, Walldius G, Van Hemelrijck M. Lipid profiles and the risk of endometrial cancer in the Swedish AMORIS study. *Int J Mol Epidemiol Genet.* 2012;3:122-133.

31. Shafique K, McLoone P, Qureshi K, Leung H, Hart C, Morrison DS. Cholesterol and the risk of grade-specific prostate cancer incidence: evidence from two large prospective cohort studies with up to 37 years' follow up. *BMC Cancer.* 2012;12:25.

32. Strohmaier S, Edlinger M, Manjer J, Stocks T, Bjorge T, Borena W, Haggstrom C, Engeland A, Nagel G, Almquist M, Selmer R, Tretli S, Concin H, Hallmans G, Jonsson H, Stattin P, Ulmer H. Total serum cholesterol and cancer incidence in the Metabolic syndrome and Cancer Project (Me-Can). *PLoS One.* 2013;8:e54242.

33. Van Hemelrijck M, Garmo H, Holmberg L, Walldius G, Jungner I, Hammar N, Lambe M. Prostate cancer risk in the Swedish AMORIS study: the interplay among triglycerides, total cholesterol, and glucose. *Cancer.* 2011;117:2086-2095.

34. Van Hemelrijck M, Walldius G, Jungner I, Hammar N, Garmo H, Binda E, Hayday A, Lambe M, Holmberg L. Low levels of apolipoprotein A-I and HDL are associated with risk of prostate cancer in the Swedish AMORIS study. *Cancer Causes Control.* 2011;22:1011-1019.

35. Adams CD, Richmond R, Ferreira DLS, Spiller W, Tan V, Zheng J, Wurtz P, Donovan J, Hamdy F, Neal D, Lane JA, Smith GD, Relton C, Eeles RA, Haiman CA, Kote-Jarai Z, Schumacher FR, Olama AAA, Benlloch S, Muir K, Berndt SI, Conti DV, Wiklund F, Chanock SJ, Gapstur S, Stevens VL, Tangen CM, Batra J, Clements JA, Gronberg H, Pashayan N, Schleutker J, Albanes D, Wolk A, West CML, Mucci LA, Cancel-Tassin G, Koutros S, Sorensen KD, Maehle L, Travis RC, Hamilton RJ, Ingles SA, Rosenstein BS, Lu YJ, Giles GG, Kibel AS, Vega A, Kogevinas M, Penney KL, Park JY, Stanford JL, Cybulski C, Nordestgaard BG, Brenner H, Maier C, Kim J, John EM, Teixeira MR, Neuhausen SL, De Ruyck K, Razack A, Newcomb LF, Lessel D, Kaneva RP, Usmani N, Claessens F, Townsend PA, Dominguez MG, Roobol MJ, Menegaux F, Khaw KT, Cannon-Albright LA, Pandha H, Thibodeau SN, Martin RM, consortium P. Circulating Metabolic Biomarkers of Screen-Detected Prostate Cancer in the ProtecT Study. *Cancer Epidemiol Biomarkers Prev.* 2019;28:208-216.

36. Kettunen J, Demirkan A, Wurtz P, Draisma HH, Haller T, Rawal R, Vaarhorst A, Kangas AJ, Lyytikainen LP, Pirinen M, Pool R, Sarin AP, Soininen P, Tukiainen T, Wang Q, Tiainen M, Tynkkynen T, Amin N, Zeller T, Beekman M, Deelen J, van Dijk KW, Esko T, Hottenga JJ, van Leeuwen EM, Lehtimaki T, Mihailov E, Rose RJ, de Craen AJ, Gieger C, Kahonen M, Perola M, Blankenberg S, Savolainen MJ, Verhoeven A, Viikari J, Willemsen G, Boomsma DI, van Duijn CM, Eriksson J, Jula A, Jarvelin MR, Kaprio J, Metspalu A, Raitakari O, Salomaa V, Slagboom PE, Waldenberger M, Ripatti S, Ala-Korpela M. Genome-wide study for circulating metabolites identifies 62 loci and reveals novel systemic effects of LPA. *Nature communications.* 2016;7:11122.

37. Kote-Jarai Z, Easton DF, Stanford JL, Ostrander EA, Schleutker J, Ingles SA, Schaid D, Thibodeau S, Dork T, Neal D, Donovan J, Hamdy F, Cox A, Maier C, Vogel W, Guy M, Muir K, Lophatananon A, Kedda MA, Spurdle A, Steginga S, John EM, Giles G, Hopper J, Chappuis PO, Hutter P, Foulkes WD, Hamel N, Salinas CA, Koopmeiners JS, Karyadi DM, Johanneson B, Wahlfors T, Tammela TL, Stern MC, Corral R, McDonnell SK, Schurmann P, Meyer A, Kuefer R, Leongamornlert DA, Tymrakiewicz M, Liu JF, O'Mara T, Gardiner RA, Aitken J, Joshi AD, Severi G, English DR, Southey M, Edwards SM, Al Olama AA, Consortium P, Eeles RA. Multiple novel prostate cancer predisposition loci confirmed by an international study: the PRACTICAL Consortium. *Cancer Epidemiol Biomarkers Prev.* 2008;17:2052-2061.

38. Bull CJ, Bonilla C, Holly JM, Perks CM, Davies N, Haycock P, Yu OH, Richards JB, Eeles R, Easton D, Kote-Jarai Z, Amin Al Olama A, Benlloch S, Muir K, Giles GG, MacInnis RJ, Wiklund F, Gronberg H, Haiman CA, Schleutker J, Nordestgaard BG, Travis RC, Neal D, Pashayan N, Khaw KT, Stanford JL, Blot WJ, Thibodeau S, Maier C, Kibel AS, Cybulski C, Cannon-Albright L, Brenner H, Park J, Kaneva R, Batra J, Teixeira MR, Micheal A, Pandha H, Smith GD, Lewis SJ, Martin RM, consortium P. Blood lipids and prostate cancer: a Mendelian randomization analysis. *Cancer Med.* 2016;5:1125-1136.

39. Do R, Willer CJ, Schmidt EM, Sengupta S, Gao C, Peloso GM, Gustafsson S, Kanoni S, Ganna A, Chen J, Buchkovich ML, Mora S, Beckmann JS, Bragg-Gresham JL, Chang HY, Demirkan A, Den Hertog HM, Donnelly LA, Ehret GB, Esko T, Feitosa MF, Ferreira T, Fischer K, Fontanillas P, Fraser RM, Freitag DF, Gurdasani D, Heikkila K, Hypponen E, Isaacs A, Jackson AU, Johansson A, Johnson T, Kaakinen M, Kettunen J, Kleber ME, Li X, Luan J, Lyytikainen LP, Magnusson PK, Mangino M, Mihailov E, Montasser ME, Muller-Nurasyid M, Nolte IM, O'Connell JR, Palmer CD, Perola M, Petersen AK, Sanna S, Saxena R, Service SK, Shah S, Shungin D, Sidore C, Song C, Strawbridge RJ, Surakka I, Tanaka T, Teslovich TM, Thorleifsson G, Van den Herik EG, Voight BF, Volcik KA, Waite LL, Wong A, Wu Y, Zhang W, Absher D, Asiki G, Barroso I, Been LF, Bolton JL, Bonnycastle LL, Brambilla P, Burnett MS, Cesana G, Dimitriou M, Doney AS, Doring A, Elliott P, Epstein SE, Eyjolfsson GI, Gigante B, Goodarzi MO, Grallert H, Gravito ML, Groves CJ, Hallmans G, Hartikainen AL, Hayward C, Hernandez D, Hicks AA, Holm H, Hung YJ, Illig T, Jones MR, Kaleebu P, Kastelein JJ, Khaw KT, Kim E, Klopp N, Komulainen P, Kumari M, Langenberg C, Lehtimaki T, Lin SY, Lindstrom J, Loos RJ, Mach F, McArdle WL, Meisinger C, Mitchell BD, Muller G, Nagaraja R, Narisu N, Nieminen TV, Nsubuga RN, Olafsson I, Ong KK, Palotie A, Papamarkou T, Pomilla C, Pouta A, Rader DJ, Reilly MP, Ridker PM, Rivadeneira F, Rudan I, Ruokonen A, Samani N, Scharnagl H, Seeley J, Silander K, Stancakova A, Stirrups K, Swift AJ, Tiret L, Uitterlinden AG, van Pelt LJ, Vedantam S, Wainwright N, Wijmenga C, Wild SH, Willemsen G, Wilsgaard T, Wilson JF, Young EH, Zhao JH, Adair LS, Arveiler D, Assimes TL, Bandinelli S, Bennett F, Bochud M, Boehm BO, Boomsma DI, Borecki IB, Bornstein SR, Bovet P, Burnier M, Campbell H, Chakravarti A, Chambers JC, Chen YD, Collins FS, Cooper RS, Danesh J, Dedoussis G, de Faire U, Feranil AB, Ferrieres J, Ferrucci L, Freimer NB, Gieger C, Groop LC, Gudnason V, Gyllensten U, Hamsten A, Harris TB, Hingorani A, Hirschhorn JN, Hofman A, Hovingh GK, Hsiung CA, Humphries SE, Hunt SC, Hveem K, Iribarren C, Jarvelin MR, Jula A, Kahonen M, Kaprio J, Kesaniemi A, Kivimaki M, Kooner JS, Koudstaal PJ, Krauss RM, Kuh D, Kuusisto J, Kyvik KO, Laakso M, Lakka TA, Lind L, Lindgren CM, Martin NG, Marz W, McCarthy MI, McKenzie CA, Meneton P, Metspalu A, Moilanen L, Morris AD, Munroe PB, Njolstad I, Pedersen NL, Power C, Pramstaller PP, Price JF, Psaty BM, Quertermous T, Rauramaa R, Saleheen D, Salomaa V, Sanghera DK, Saramies J, Schwarz PE, Sheu WH, Shuldiner AR, Siegbahn A, Spector TD, Stefansson K, Strachan DP, Tayo BO, Tremoli E, Tuomilehto J, Uusitupa M, van Duijn CM, Vollenweider P, Wallentin L, Wareham NJ, Whitfield JB, Wolffenbuttel BH, Altshuler D, Ordovas JM, Boerwinkle E, Palmer CN, Thorsteinsdottir U, Chasman DI, Rotter JI, Franks PW, Ripatti S, Cupples LA, Sandhu MS, Rich SS, Boehnke M, Deloukas P, Mohlke KL, Ingelsson E, Abecasis GR, Daly MJ, Neale BM, Kathiresan S. Common variants associated with plasma triglycerides and risk for coronary artery disease. *Nat Genet.* 2013;45:1345-1352.

40. Teslovich TM, Musunuru K, Smith AV, Edmondson AC, Stylianou IM, Koseki M, Pirruccello JP, Ripatti S, Chasman DI, Willer CJ, Johansen CT, Fouchier SW, Isaacs A, Peloso GM, Barbalic M, Ricketts SL, Bis JC, Aulchenko YS, Thorleifsson G, Feitosa MF, Chambers J, Orho-Melander M, Melander O, Johnson T, Li X, Guo X, Li M, Shin Cho Y, Jin Go M, Jin Kim Y, Lee JY, Park T, Kim K, Sim X, Twee-Hee Ong R, Croteau-Chonka DC, Lange LA, Smith JD, Song K, Hua Zhao J, Yuan X, Luan J, Lamina C, Ziegler A, Zhang W, Zee RY, Wright AF, Witteman JC, Wilson JF, Willemsen G, Wichmann HE, Whitfield JB, Waterworth DM, Wareham NJ, Waeber G, Vollenweider P, Voight BF, Vitart V, Uitterlinden AG, Uda M, Tuomilehto J, Thompson JR, Tanaka T, Surakka I, Stringham HM, Spector TD, Soranzo N, Smit JH, Sinisalo J, Silander K, Sijbrands EJ, Scuteri A, Scott J, Schlessinger D, Sanna S, Salomaa V, Saharinen J, Sabatti C, Ruokonen A, Rudan I, Rose LM, Roberts R, Rieder M, Psaty BM, Pramstaller PP, Pichler I, Perola M, Penninx BW, Pedersen NL, Pattaro C, Parker AN, Pare G, Oostra BA, O'Donnell CJ, Nieminen MS, Nickerson DA, Montgomery GW, Meitinger T, McPherson R, McCarthy MI, McArdle W, Masson D, Martin NG, Marroni F, Mangino M, Magnusson PK, Lucas G, Luben R, Loos RJ, Lokki ML, Lettre G, Langenberg C, Launer LJ, Lakatta EG, Laaksonen R, Kyvik KO, Kronenberg F, Konig IR, Khaw KT, Kaprio J, Kaplan LM, Johansson A, Jarvelin MR, Janssens AC, Ingelsson E, Igl W, Kees Hovingh G, Hottenga JJ, Hofman A, Hicks AA, Hengstenberg C, Heid IM, Hayward C, Havulinna AS, Hastie ND, Harris TB, Haritunians T, Hall AS, Gyllensten U, Guiducci C, Groop LC, Gonzalez E, Gieger C, Freimer NB, Ferrucci L, Erdmann J, Elliott P, Ejebe KG, Doring A, Dominiczak AF, Demissie S, Deloukas P, de Geus EJ, de Faire U, Crawford G, Collins FS, Chen YD, Caulfield MJ, Campbell H, Burtt NP, Bonnycastle LL, Boomsma DI, Boekholdt SM, Bergman RN, Barroso I, Bandinelli S, Ballantyne CM, Assimes TL, Quertermous T, Altshuler D, Seielstad M, Wong TY, Tai ES, Feranil AB, Kuzawa CW, Adair LS, Taylor HA, Jr., Borecki IB, Gabriel SB, Wilson JG, Holm H, Thorsteinsdottir U, Gudnason V, Krauss RM, Mohlke KL, Ordovas JM, Munroe PB, Kooner JS, Tall AR, Hegele RA, Kastelein JJ, Schadt EE, Rotter JI, Boerwinkle E, Strachan DP, Mooser V, Stefansson K, Reilly MP, Samani NJ, Schunkert H, Cupples LA, Sandhu MS, Ridker PM, Rader DJ, van Duijn CM, Peltonen L, Abecasis GR, Boehnke M, Kathiresan S. Biological, clinical and population relevance of 95 loci for blood lipids. *Nature.* 2010;466:707-713.

41. Willer CJ, Schmidt EM, Sengupta S, Peloso GM, Gustafsson S, Kanoni S, Ganna A, Chen J, Buchkovich ML, Mora S, Beckmann JS, Bragg-Gresham JL, Chang HY, Demirkan A, Den Hertog HM, Do R, Donnelly LA, Ehret GB, Esko T, Feitosa MF, Ferreira T, Fischer K, Fontanillas P, Fraser RM, Freitag DF, Gurdasani D, Heikkila K, Hypponen E, Isaacs A, Jackson AU, Johansson A, Johnson T, Kaakinen M, Kettunen J, Kleber ME, Li X, Luan J, Lyytikainen LP, Magnusson PKE, Mangino M, Mihailov E, Montasser ME, Muller-Nurasyid M, Nolte IM, O'Connell JR, Palmer CD, Perola M, Petersen AK, Sanna S, Saxena R, Service SK, Shah S, Shungin D, Sidore C, Song C, Strawbridge RJ, Surakka I, Tanaka T, Teslovich TM, Thorleifsson G, Van den Herik EG, Voight BF, Volcik KA, Waite LL, Wong A, Wu Y, Zhang W, Absher D, Asiki G, Barroso I, Been LF, Bolton JL, Bonnycastle LL, Brambilla P, Burnett MS, Cesana G, Dimitriou M, Doney ASF, Doring A, Elliott P, Epstein SE, Ingi Eyjolfsson G, Gigante B, Goodarzi MO, Grallert H, Gravito ML, Groves CJ, Hallmans G, Hartikainen AL, Hayward C, Hernandez D, Hicks AA, Holm H, Hung YJ, Illig T, Jones MR, Kaleebu P, Kastelein JJP, Khaw KT, Kim E, Klopp N, Komulainen P, Kumari M, Langenberg C, Lehtimaki T, Lin SY, Lindstrom J, Loos RJF, Mach F, McArdle WL, Meisinger C, Mitchell BD, Muller G, Nagaraja R, Narisu N, Nieminen TVM, Nsubuga RN, Olafsson I, Ong KK, Palotie A, Papamarkou T, Pomilla C, Pouta A, Rader DJ, Reilly MP, Ridker PM, Rivadeneira F, Rudan I, Ruokonen A, Samani N, Scharnagl H, Seeley J, Silander K, Stancakova A, Stirrups K, Swift AJ, Tiret L, Uitterlinden AG, van Pelt LJ, Vedantam S, Wainwright N, Wijmenga C, Wild SH, Willemsen G, Wilsgaard T, Wilson JF, Young EH, Zhao JH, Adair LS, Arveiler D, Assimes TL, Bandinelli S, Bennett F, Bochud M, Boehm BO, Boomsma DI, Borecki IB, Bornstein SR, Bovet P, Burnier M, Campbell H, Chakravarti A, Chambers JC, Chen YI, Collins FS, Cooper RS, Danesh J, Dedoussis G, de Faire U, Feranil AB, Ferrieres J, Ferrucci L, Freimer NB, Gieger C, Groop LC, Gudnason V, Gyllensten U, Hamsten A, Harris TB, Hingorani A, Hirschhorn JN, Hofman A, Hovingh GK, Hsiung CA, Humphries SE, Hunt SC, Hveem K, Iribarren C, Jarvelin MR, Jula A, Kahonen M, Kaprio J, Kesaniemi A, Kivimaki M, Kooner JS, Koudstaal PJ, Krauss RM, Kuh D, Kuusisto J, Kyvik KO, Laakso M, Lakka TA, Lind L, Lindgren CM, Martin NG, Marz W, McCarthy MI, McKenzie CA, Meneton P, Metspalu A, Moilanen L, Morris AD, Munroe PB, Njolstad I, Pedersen NL, Power C, Pramstaller PP, Price JF, Psaty BM, Quertermous T, Rauramaa R, Saleheen D, Salomaa V, Sanghera DK, Saramies J, Schwarz PEH, Sheu WH, Shuldiner AR, Siegbahn A, Spector TD, Stefansson K, Strachan DP, Tayo BO, Tremoli E, Tuomilehto J, Uusitupa M, van Duijn CM, Vollenweider P, Wallentin L, Wareham NJ, Whitfield JB, Wolffenbuttel BHR, Ordovas JM, Boerwinkle E, Palmer CNA, Thorsteinsdottir U, Chasman DI, Rotter JI, Franks PW, Ripatti S, Cupples LA, Sandhu MS, Rich SS, Boehnke M, Deloukas P, Kathiresan S, Mohlke KL, Ingelsson E, Abecasis GR, Global Lipids Genetics C. Discovery and refinement of loci associated with lipid levels. *Nat Genet.* 2013;45:1274-1283.

42. Isaacs A, Willems SM, Bos D, Dehghan A, Hofman A, Ikram MA, Uitterlinden AG, Oostra BA, Franco OH, Witteman JC, van Duijn CM. Risk scores of common genetic variants for lipid levels influence atherosclerosis and incident coronary heart disease. *Arterioscler Thromb Vasc Biol.* 2013;33:2233-2239.

43. Orho-Melander M, Hindy G, Borgquist S, Schulz CA, Manjer J, Melander O, Stocks T. Blood lipid genetic scores, the HMGCR gene and cancer risk: a Mendelian randomization study. *Int J Epidemiol.* 2018;47:495-505.

44. Beeghly-Fadiel A, Khankari NK, Delahanty RJ, Shu XO, Lu Y, Schmidt MK, Bolla MK, Michailidou K, Wang Q, Dennis J, Yannoukakos D, Dunning AM, Pharoah PDP, Chenevix-Trench G, Milne RL, Hunter DJ, Per H, Kraft P, Simard J, Easton DF, Zheng W. A Mendelian randomization analysis of circulating lipid traits and breast cancer risk. *Int J Epidemiol.* 2020;49:1117-1131.

45. Johnson KE, Siewert KM, Klarin D, Damrauer SM, Program VAMV, Chang KM, Tsao PS, Assimes TL, Maxwell KN, Voight BF. The relationship between circulating lipids and breast cancer risk: A Mendelian randomization study. *PLoS Med.* 2020;17:e1003302.

46. Nowak C, Arnlov J. A Mendelian randomization study of the effects of blood lipids on breast cancer risk. *Nature communications.* 2018;9:3957.

47. Kho PF, Amant F, Annibali D, Ashton K, Attia J, Auer PL, Beckmann MW, Black A, Brinton L, Buchanan DD, Chanock SJ, Chen C, Chen MM, Cheng THT, Cook LS, Crous-Bous M, Czene K, De Vivo I, Dennis J, Dork T, Dowdy SC, Dunning AM, Durst M, Easton DF, Ekici AB, Fasching PA, Fridley BL, Friedenreich CM, Garcia-Closas M, Gaudet MM, Giles GG, Goode EL, Gorman M, Haiman CA, Hall P, Hankinson SE, Hein A, Hillemanns P, Hodgson S, Hoivik EA, Holliday EG, Hunter DJ, Jones A, Kraft P, Krakstad C, Lambrechts D, Le Marchand L, Liang X, Lindblom A, Lissowska J, Long J, Lu L, Magliocco AM, Martin L, McEvoy M, Milne RL, Mints M, Nassir R, Otton G, Palles C, Pooler L, Proietto T, Rebbeck TR, Renner SP, Risch HA, Rubner M, Runnebaum I, Sacerdote C, Sarto GE, Schumacher F, Scott RJ, Setiawan VW, Shah M, Sheng X, Shu XO, Southey MC, Tham E, Tomlinson I, Trovik J, Turman C, Tyrer JP, Van Den Berg D, Wang Z, Wentzensen N, Xia L, Xiang YB, Yang HP, Yu H, Zheng W, Webb PM, Thompson DJ, Spurdle AB, Glubb DM, O'Mara TA. Mendelian randomization analyses suggest a role for cholesterol in the development of endometrial cancer. *Int J Cancer.* 2021;148:307-319.
